# A 10-Gene Signature Identified by Machine Learning for Predicting the Response to Transarterial Chemoembolization in Patients with Hepatocellular Carcinoma

**DOI:** 10.1155/2022/3822773

**Published:** 2022-01-24

**Authors:** Yiyang Tang, Yanqin Wu, Miao Xue, Bowen Zhu, Wenzhe Fan, Jiaping Li

**Affiliations:** Department of Interventional Oncology, The First Affiliated Hospital of Sun Yat-Sen University, Guangzhou 510080, China

## Abstract

**Background:**

Transarterial chemoembolization (TACE) is recommended for intermediate-stage HCC patients. Owing to substantial variation in its efficacy, indicators of patient responses to TACE need to be determined.

**Methods:**

A Gene Expression Omnibus (GEO) dataset consisting of patients of different TACE-response status was retrieved. Differentially expressed genes (DEGs) were calculated and variable gene ontology analyses were conducted. Potential drugs and response to immunotherapy were predicted using multiple bioinformatic algorithms. We built and compared 5 machine-learning models with finite genes to predict patients' response to TACE. The model was also externally validated to discern different survival outcomes after TACE. Tumor-infiltrating lymphocytes (TILs) and tumor stemness index were evaluated to explore potential mechanism of our model.

**Results:**

The gene set variation analysis revealed enhanced pathways related to G2/M checkpoint, E2F, mTORC1, and myc in TACE nonresponders. TACE responders had better immunotherapy response too. 373 DEGs were detected and the upregulated DEGs in nonresponders were enriched in IL-17 signal pathway. 5 machine-learning models were constructed and evaluated, and a linear support vector machine (SVM)-based model with 10 genes was selected (AQP1, FABP4, HERC6, LOX, PEG10, S100A8, SPARCL1, TIAM1, TSPAN8, and TYRO3). The model achieved an AUC and accuracy of 0.944 and 0.844, respectively, in the development cohort. In the external validation cohort comprised of patients receiving adjuvant TACE and postrecurrence TACE treatment, the predicted response group significantly outlived the predicted nonresponse counterparts. TACE nonresponders tend to have more macrophage M0 cells and lower resting mast cells in the tumor tissue and the stemness index is also higher than responders. Those characteristics were successfully captured by our model.

**Conclusion:**

The model based on expression data of 10 genes could potentially predict HCC patients' response and prognosis after TACE treatment. The discriminating power was TACE-specific.

## 1. Introduction

Transarterial chemoembolization (TACE) is recommended as the first-line therapy for intermediate-stage HCC based on the Barcelona Clinic Liver Cancer (BCLC) staging system. TACE is also used outside of intermediate HCC after recommended methods fail to achieve satisfactory results [1]. The response rate at 1 month after TACE ranges from 39.6% to 87%, with variation among studies [2–4].

Owing to the heterogeneity of intermediate-stage HCC and broad application of TACE beyond recommended settings, patient responses are highly variable. Thus, it is necessary to develop a method to select patients expected to benefit from this procedure [5]. Multiple scoring systems have been established to predict outcomes after TACE based on routinely measured biomarkers, such as the hepatic arterial embolization prognostic (HAP) score and enhanced derivatives [6, 7]. However, these models are mostly HCC-specific and not TACE-specific [8]. Recently, post-TACE transient hypertransaminase (elevation of >52% alanine aminotransferase and >46% aspartate aminotransferase from baseline) was found to be a good indicator of TACE response [9]. Thus, it is vital to develop a TACE-specific method for the selection of candidates for TACE therapy before TACE operation. The increasing clinical application of gene sequencing and accumulation of related data provide a basis for the development of a gene signature for predicting the response to TACE in precision oncology.

In this study, we evaluated associations between transcriptomic data for individual patients and the response to TACE. We employed a gene expression database from Gene Expression Omnibus (GEO) to develop a predictive gene signature for the response to TACE and validated its efficacy with an external dataset.

## 2. Material and Methods

### 2.1. Gene Expression Data Obtaining and Preprocessing

The development cohort GSE104580 (https://www.ncbi.nlm.nih.gov/geo/query/acc.cgi?acc=GSE104580) came from a continuing study based on a clinical trial under registration number https://ClinicalTrials.gov.NCT00493402. The dataset comprised 147 patients with unresectable HCCs and no significant baseline liver dysfunction. Those treatment-naïve patients received TACE as their primary treatment and 81 of them were labelled as TACE responders and 66 were marked as TACE nonresponders. The RNA was extracted from HCC patients before TACE treatment.

The gene expression as well as clinical data of external validation cohort came from GSE14520 (https://www.ncbi.nlm.nih.gov/geo/query/acc.cgi?acc=GSE14520) including 74 HCC patients receiving adjuvant TACE after liver resection, 30 patients receiving postrecurrence TACE treatment, and 85 patients receiving liver resection only. The detail of clinical information of the external validation cohort was described in previous research [10].

The gene expression profiles were retrieved from GEO database using GEOquery package in R. Probes corresponding to multiple genes or probes corresponding to default genes were discarded. Once there were multiple probes for one gene, the probes with max average expression across all samples were preserved. The gene expression data were transformed to z-scores for better extrapolation of the model.

### 2.2. Gene Set Variation Analysis (GSVA)

GSVA were adopted to discern the differentially enriched pathways between TACE nonresponders and responders. We chose the representative hallmark gene set for enrichment analysis and the whole operation was carried out using GSVA package in R.

### 2.3. Differentially Expressed Genes (DEGs) Distinguishing and Gene Ontology Analysis

To find out DEGs between response and nonresponse groups, we employed Limma package in R and set the threshold to be |log2 Fold Change| > 1 and Benjamini-Hochberg adjusted *p* < 0.05 to mark off DEGs. Subsequent Kyoto Encyclopedia of Genes and Genomes (KEGG) enrichment analysis was conducted to explore the differentially enriched pathways between 2 response statuses.

### 2.4. Protein-Protein Interaction Network (PPI) Construction

STRING database was used to infer the potential interactions between proteins encoded by DEGs. We employed Cytoscape software to visualize the PPI network. Molecular COmplex DEtection (MCODE) plugin was used to extract the highly interacted subnetwork within the whole PPI.

### 2.5. Potential Compounds Detection

We searched the Connectivity Map (CMap) database (https://clue.io/cmap) for potential chemicals which could elicit opposite transcriptomic alterations as we observed in the nonresponse group compared with the response group. CMap is a genome-scale library of cellular signatures storing the response to chemical, genetic, and disease perturbation [11]. By comparing the transcriptomic change in our samples with those caused by related perturbagens collected in the library, the CMap could predict drugs with their annotated mode of action (MoA). In this research, we queried CMap build 1.0 based on L1000 assay with DEGs between TACE responders and nonresponders and counted those compounds whose connectivity scores associated with HepG2 cell line were less than −90 as potential cure.

Meanwhile, Genomics of Drug Sensitivity in Cancer (GDSC) database stored genomic expression profiles of considerable cell lines and their drug response data measured with half-maximal inhibitory concentration (IC_50_). The GDSC consist of 2 databases; GDSC1 contained 958 cell lines and 367 drugs while GDSC2 contained 805 cell lines and 198 drugs. We utilized the data from GDSC to speculate the response to different drugs using oncoPredict package in R [12].

Besides, the Tumor Immune Dysfunction and Exclusion (TIDE, https://tide.dfci.harvard.edu/) algorithm was employed to deduce sample's response to immunotherapy. TIDE was a framework developed to use gene expression profile to assess the potential of tumor immune evasion and thus predict response to immune checkpoint blockade such as anti-PD1 (programmed cell death protein 1) and anti-CTLA4 (cytotoxic T-lymphocyte-associated protein 4).

### 2.6. Machine-Learning-Based Gene Selection

Our study applied 5 commonly used models including least absolute shrinkage and selection operator (Lasso) logistic regression, linear support vector machine (SVM), artificial neural network (ANN), random forest, and eXtreme Gradient Boosting (XGBoost)-based tree model. 147 patients from GSE104580 with their DEGs expression data composed the development cohort. In each iteration, the development was randomly split into 80% training cohort and 20% testing cohort. Only the training cohort was used to generate the model. We tracked the AUC, accuracy, F1 score, Youden index, sensitivity, specificity, positive predictive value (PPV), and negative predictive value (NPV). In addition, we calculated the weight given to each gene involved in constructing the model and ranked those genes according to their importance. Once a gene was ranked top 20% among all DEGs, we marked it as one occurrence. After 10 times of iterations, those genes with occurrence no less than 8 were preserved for further model construction.

The weights of genes were returned by the model directly through coefficient or feature importance attributions, except for ANN model. Therefore, the mean absolute SHAP (Shapley additive explanation) values across all the test sets were employed to determine the importance of each gene in the ANN model. In this method, the SHAP value of each feature indicates the contribution it made to build up the result. The processes were carried out using SHAP package in Python.

As for the tuning of hyperparameters for each model, 5-fold grid search cross validation was used to optimizing the hyperparameters for Lasso-logistic regression, SVM, and ANN, while we adopt Bayesian optimization to tune the hyperparameters for random forest and XGBoost-based tree model to accelerate the training speed. AUC was employed as the main metric to assess the performance of the model.

### 2.7. Establishment of Gene Signature and External Validation

Those genes whose occurrence exceeded 8 and were simultaneously present in GSE14520 were selected to construct the gene signature for each model, respectively. Model was built and evaluated in the development cohort with 20% of the data chosen as test set. During 10 rounds construction-evaluation loop, the AUC, accuracy, F1 score, Youden index, sensitivity, specificity, PPV, and NPV were recorded and averaged to improve the validity of model selection. Subsequent model with highest AUC was chosen as the best-performed model to predict the response status of each sample in the external validation cohort.

### 2.8. Tumor-Infiltrating Lymphocytes (TILs) Evaluation

CIBERSORTx is a widely used algorithm which could approximate the cell composition of bulk tissues. The results were verified to be highly consistent with truth [13]. To assess the different fractions of TILs, we utilized LM22 signature matrix in CIBERSORTx to calculate the proportions of 22 subtypes of immune cells with 1000 permutations.

### 2.9. Tumor Stemness Evaluation

We adopted the algorithm presented earlier and developed the one-class logistic regression machine-learning model (OCLR) trained on expression profiles of a collection of stem cells from PCBC database (https://www.synapse.org/#!Synapse:syn1773109) using GELnet package in R [14]. We used the summarized normalized mRNA matrix (syn2701943) of those cells labelled as SC (stem cell) only. The model was constructed using leave-one-out cross-validation technique. After the establishment of the stemness signature, we scored the mRNA stemness index (mRNAsi) of a new sample through calculating the spearman correlation between the expression data of sample and the model's weight of related genes. The correlation coefficients were later transformed through min-max standardization for better interpretation.

### 2.10. Statistics

Statistical analyses in this research were performed using associated package in Python and R. OS and RFS curves were drawn with Kaplan–Meier method and difference in survival results were evaluated with log-rank test. Univariate and multivariate Cox regression models were employed to identify valuable feature to predict the prognosis of patients. Independent *t*-test was implemented to detect any divergence between groups. All statistical tests were two-tailed, and we considered *p* < 0.05 a significant result.

## 3. Results

### 3.1. Enriched Pathways for Differentially Expressed Genes between TACE Responders and Nonresponders

**Figure 1:**
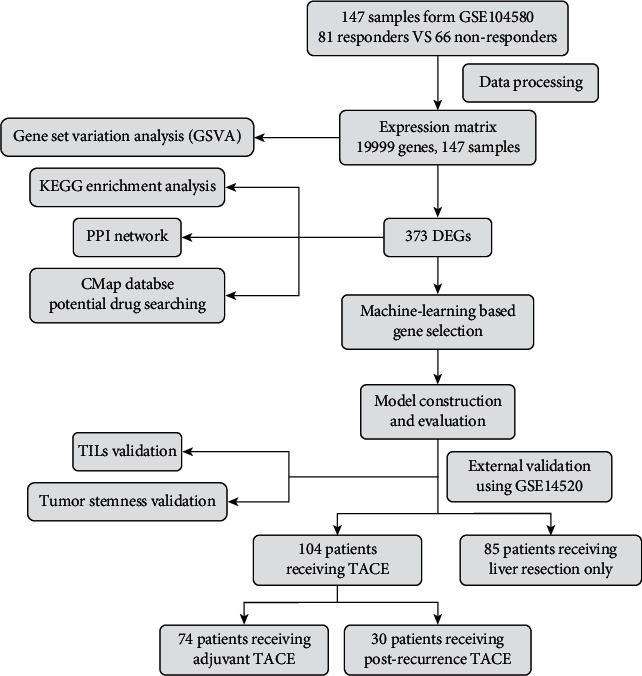
Flowchart displaying detailed process of this research.

After processing microarray data, we obtained an expression matrix with 147 samples and 19999 genes. Considering the heterogeneity in the response to TACE, we performed a GSVA for each sample and identified marked pathway enrichment for the comparison between TACE responders and nonresponders. The detailed procedure is shown in Figure 1.

**Figure 2:**
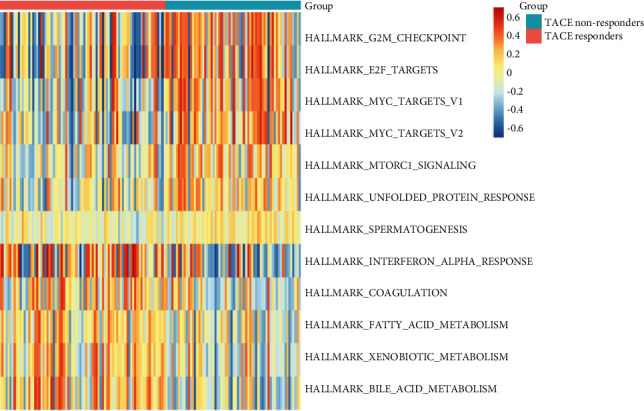
Significant differentially enriched pathways between TACE responders and nonresponders. The TOP7 pathways were remarkably upregulated among TACE nonresponders while the bottom 5 pathways were abnormally enriched in TACE responders.

Widely used and representative hallmark gene sets, including 50 carefully curated gene sets summarizing the main biological states and processes, were employed for further analyses. We identified seven highly enriched and five poorly enriched gene sets in TACE nonresponders compared to responders. As shown in Figure 2, in nonresponders, genes downstream of mammalian target of rapamycin complex 1 (mTORC1), E2F, and MYC as well as genes related to the G2/M checkpoint, unfolded protein response, and spermatogenesis were upregulated. Genes associated with the interferon *α* response, coagulation, fatty acid, xenobiotics, and bile acid metabolism were remarkedly downregulated.

### 3.2. Differentially Expressed Genes between TACE Responders and Nonresponders

**Figure 3:**
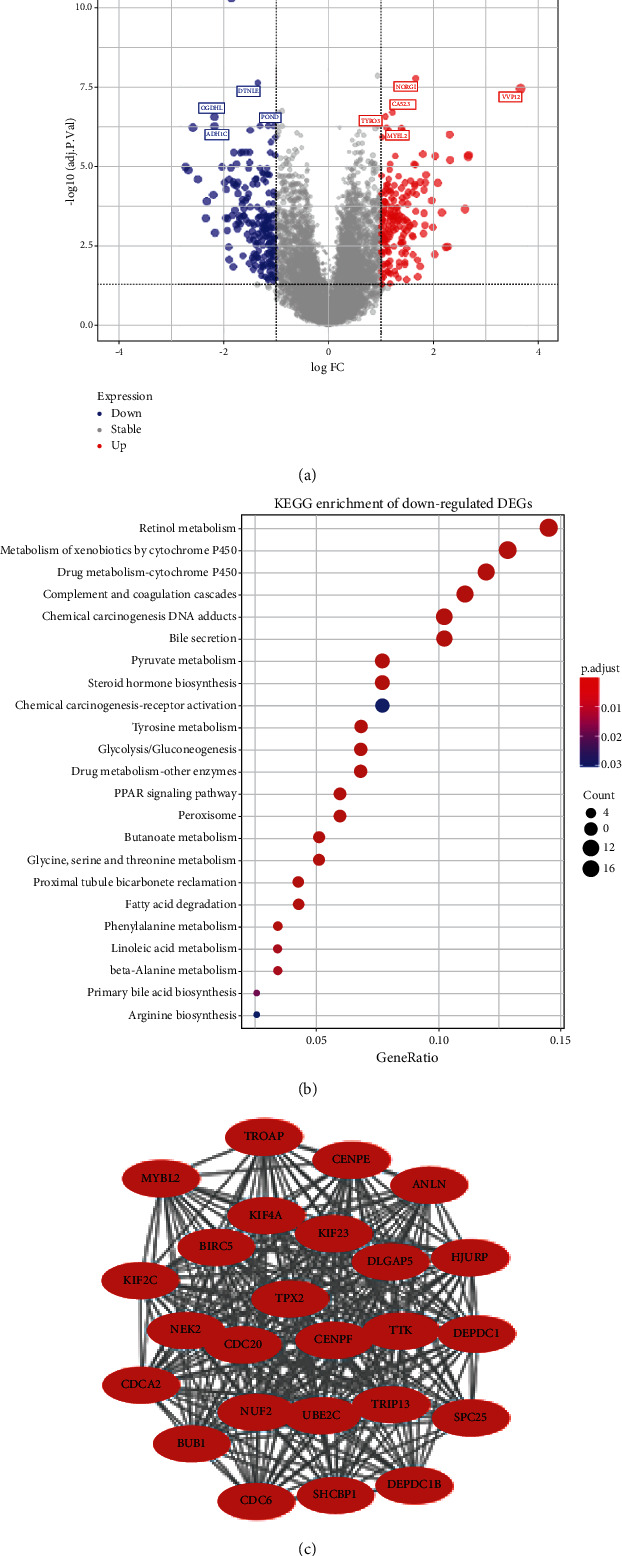
Analysis of DEGs. (a) Volcano plot displaying the distribution of 373 detected DEGs. Red dots indicate overexpressed genes in TACE nonresponders compared with responders while blue dots denoted the downregulated genes. The top 5 upregulated and downregulated genes were labelled with symbol on the plot. |log2 Fold Change| > 1 and Benjamini-Hochberg adjusted *p* < 0.05 was chosen as the threshold. (b) KEGG enrichment analysis of downregulated DEGs in nonresponders. The color of dots indicates different *p* values while the size implies the number of genes enriched in the given set. (c) Extracted PPI subnetwork using MCODE plugin in Cytoscape software. The red color implies an upregulated status in nonresponders.

By comparing gene expression levels between 81 TACE nonresponders and 66 TACE responders, we screened out 373 DEGs, among which 179 were upregulated and 194 were downregulated in nonresponders (Figure 3(a), Supplementary Table 1). In a KEGG analysis, the IL-17 signaling pathway was the only enriched pathway for upregulated DEGs, while 23 pathways were significantly enriched for downregulated DEGs (Figure 3(b)).

We input these DEGs into STRING and constructed a PPI network with 303 nodes and 1319 edges. Within this network, we extracted the subnetwork with the most interactions, including 25 upregulated genes and 291 edges (Figure 3(c)). A KEGG analysis revealed that these genes are highly enriched in cell cycle pathways. We also adopted the CMap database to identify potential compounds that could offset the dysregulation of DEGs in TACE nonresponders.

**Figure 4:**
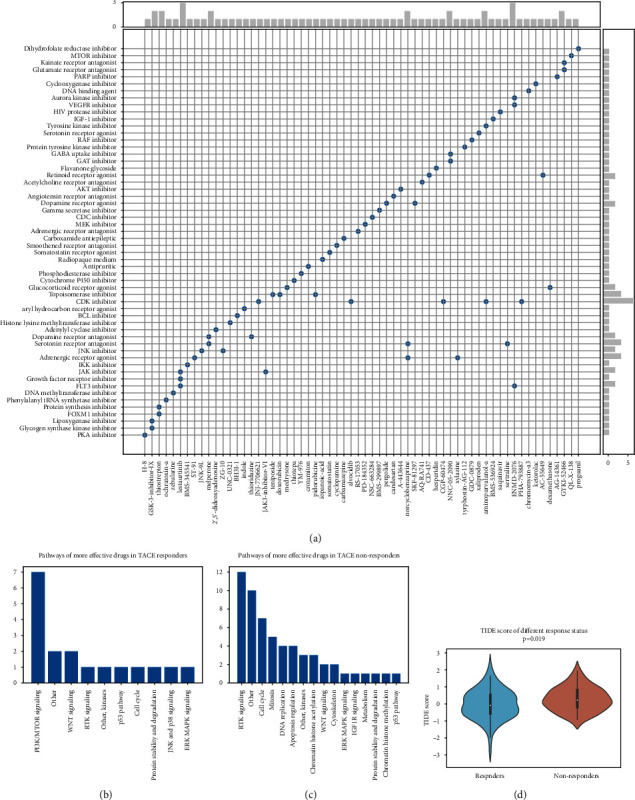
Drug sensitivity prediction. (a) Compounds predicted by cMAP to attenuate the dysregulation of genes observed in TACE nonresponders. The *x*- and *y*-axis labels correspond to the name and category of the compounds, respectively. The microhistograms on the top and right summarize related frequencies. (b, c) Summary of enriched pathways of drugs which are more effective in TACE responders and nonresponders. (d) TIDE score of TACE responders and nonresponders; lower TIDE score indicated better response to immunotherapy.

We specifically focused on reagents that could elicit opposite responses in the well-known human HCC cell line HepG2. As shown in Figure 4(a), 62 chemicals with connectivity scores of less than −90 were detected. Their MoAs were recorded and CDK inhibitor was identified as the best candidate to reverse nonresponse status. It was noteworthy that the commonly used doxorubicin and pidorubicine which is the synonym of epirubicin were potential reagents which could treat the TACE nonresponse status. Besides, we also applied oncoPredict package to infer the drug response status with genomic expression data. Combining results from GDSC1 and GDSC2, we detected 18 and 58 drugs which were more effective in response and nonresponse groups, respectively (Supplementary Tables 2 and 3). It should be noted cisplatin was ranked high in those effective drugs in nonresponders. As sorted and presented in Figures 4(b)–4(c), most effective drugs in TACE responders belong to PI3K/MTOR signaling pathway, while in TACE nonresponders, most drugs belong to RTK signaling pathway. Additionally, we also employed TIDE algorithm to predict samples' response to thriving immunotherapy. Since lower TIDE score indicates more better response to immunotherapy, the result in Figure 4(d) suggested that TACE responders were more likely to benefit from immunotherapy.

### 3.3. Establishment of a Gene Signature

For the development of predictive gene signatures for the response to TACE, we compared five common machine-learning models, including Lasso-logistic regression, linear SVM, random forest, XGBoost, and artificial neural networks. The model was developed using a training cohort and evaluated using an internal validation cohort, and the importance of each gene was recorded. During 10 rounds of replication, we tracked genes included in model construction and ranked genes based on the importance coefficient returned by the model. We recorded genes in the top 74 (20% of 373 DEGs) in each replication and selected those genes obtained in at least 8 of 10 rounds for further analyses. The performance of each model based on 373 DEGs is shown in Supplementary Table 4. The top 20 important genes of each model are listed in Supplementary Table 5. We evaluated the AUC, F1 score, accuracy, Youden index, sensitivity, specificity, PPV, and NPV for each model.

**Figure 5:**
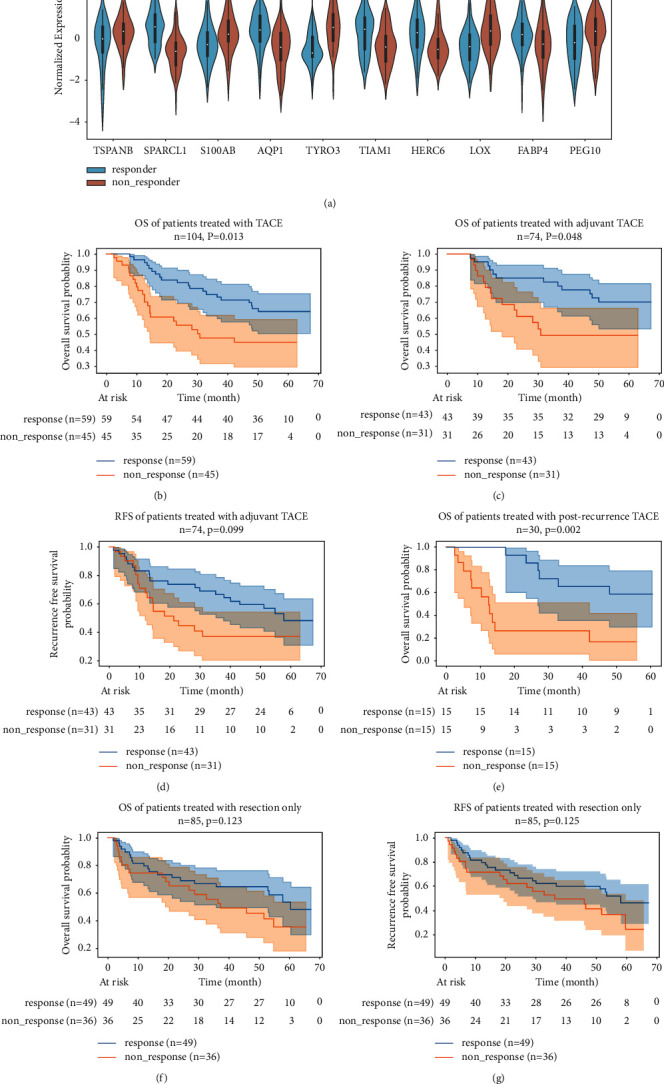
Survival analysis of external validation cohort. (a) Violin plot demonstrating the expression level of 10 genes of interest in TACE responders and nonresponders. (b) OS of predicted response and nonresponse HCC patients receiving both adjuvant and postrecurrence TACE treatment. (c) OS of patients receiving adjuvant TACE treatment. (d) RFS of different group of patients receiving adjuvant TACE treatment. (e) OS of patients receiving postrecurrence TACE treatment. (f) OS of patients receiving liver resection only. (g) RFS of patients receiving liver resection only.

Next, we selected intersected genes that were also present in GSE14520 to avoid overfitting and to facilitate external validation. We retrained and verified the efficacy of different models within the development cohort. As summarized in Table 1, after 10 rounds of repetition, the SVM achieved the highest average AUC score and was chosen for further analyses. After applying it to the full development cohort, the SVM model achieved an AUC of 0.944 and an accuracy of 0.844. The SVM model consisted of 10 genes, including aquaporin 1 (AQP1), FABP4, HECT and RLD domain-containing E3 ubiquitin protein ligase family member 6 (HERC6), lysyl oxidase (LOX), paternally expressed 10 (PEG10), S100 calcium binding protein A8 (S100A8), SPARC-like 1 (SPARCL1), TIAM Rac1 associated GEF 1 (TIAM1), Tetraspanin 8 (TSPAN8), and TYRO3 protein tyrosine kinase (TYRO3) (Table 2). The expression levels of these 10 genes in both groups are shown in Figure 5(a). TSPAN8, S100A8, TYRO3, LOX, and PEG10 were overexpressed in nonresponders, while SPARCL1, AQP1, TIAM1, HERC6, and FABP4 were expressed at low levels in nonresponders.

### 3.4. External Validation of the Gene Signature

To further test the predictive ability of our model, we chose patients from GSE14520 for external validation, including 74 patients treated with adjuvant TACE and 30 patients treated with postrecurrence TACE. In the external validation cohort, scores for each sample were calculated and samples were divided into TACE response and nonresponse groups with a threshold of 0.5. As shown in Figure 5(b), the predicted response group had a remarkably longer OS than that of the nonresponse group. In terms of patients receiving adjuvant TACE treatment after liver resection, our model successfully predicted a group of patients with a considerably longer OS (Figure 5(c)). However, our model failed to detect patients with a longer RFS after adjuvant TACE (Figure 5(d)). For patients who received postrecurrence TACE treatment, the prognosis diverged dramatically between the two groups (Figure 5(e)). To determine whether the predictive power was exclusive to TACE treatment, we applied our model to patients who received liver resection only. As shown in Figures 5(f) and 5(g), the OS and RFS values were similar in the predicted response and nonresponse groups, supporting the specificity of our model for the prediction of the TACE response. Moreover, we calculated the AUC values and generated time-dependent ROC curves for the prediction of 1-, 3-, and 5-year survival in different populations (Supplementary Figures 1A–1F). As displayed in Supplementary Figure 1G, our model achieved the best performance in predicting OS in patients receiving postrecurrence TACE.

### 3.5. Independent Prognostic Factor for the OS of TACE-Treated Patients

Combining the clinical data for patients in GSE14520, we performed univariate Cox analyses to explore the predictive value of a series of clinical metrics. As shown in Table 3, a larger main tumor size (>5 cm), higher BCLC stage, and the predicted response status by our model were identified as meaningful risk factors. We performed a multivariate Cox analysis including these three variables and found that the predicted response status of our model was an independent predictor.

### 3.6. Differences in TIL Components and Tumor Stemness between TACE Response and Nonresponse Groups

**Figure 6:**
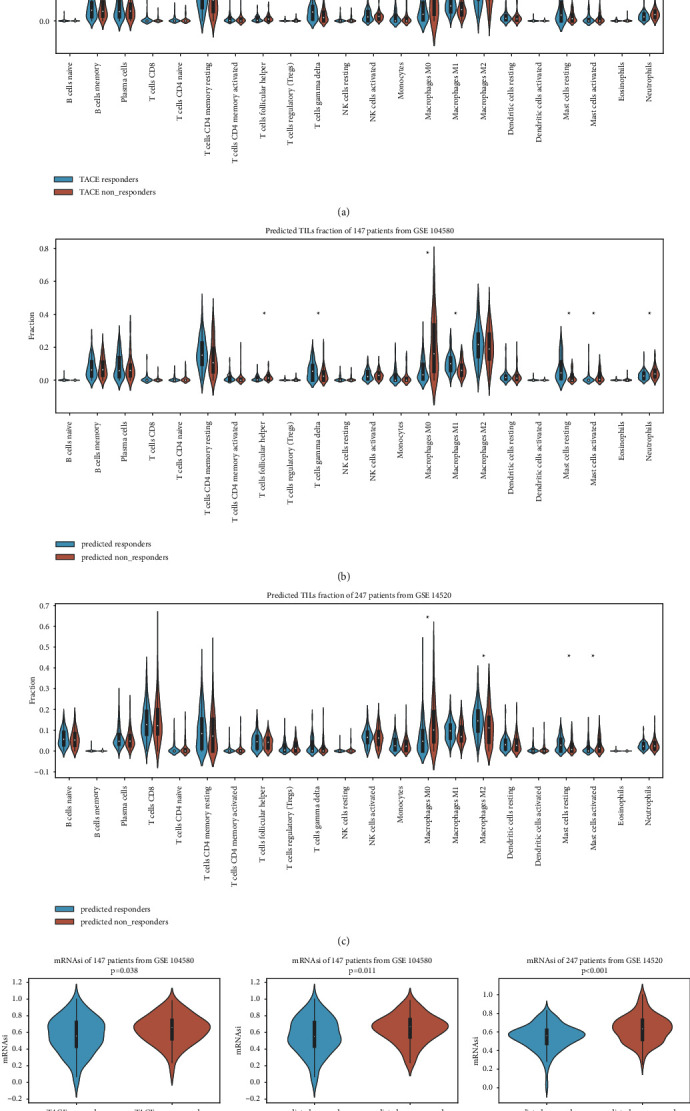
TILs and tumor stemness comparison between response and nonresponse groups. (a) Violin plot displaying 22 TILs distribution using CIBERSORTx algorithm between the given TACE responders and nonresponders in GSE104580. (b) Comparison of 22 TILs fraction in predicted responders and nonresponders in GSE104580 with our devised model. (c) Comparison of 22 TILs fraction in predicted responders and nonresponders in GSE14520 with our devised model. (d) Tumor stemness index between given TACE responders and nonresponders in GSE104580. (e) Tumor stemness index of predicted response and nonresponse patients in GSE104580 using our model. (f) Tumor stemness index of predicted response and nonresponse group in GSE14520 using our model.

The tumor microenvironment and tumor stemness are strongly associated with TACE outcomes; accordingly, we further investigated the mechanism underlying the predictive value of our model; we focused on TILs and tumor stemness [15]. We utilized the CIBERSORTx algorithm to explore the differences in proportions of TILs between TACE responders and nonresponders. As demonstrated in Figure 6(a), the TACE nonresponders tended to have remarkably more macrophage M0 cells and neutrophils with fewer *γδ*T cells, macrophage M1 cells, and resting mast cells than the responders. We also compared the predicted response and nonresponse groups in the development and validation cohorts to determine whether our model could capture these differences in immune cell infiltration. Among the five abnormally enriched cell types, the higher frequency of macrophage M0 and lower frequency of resting mast cells in TACE nonresponders were corroborated using our model within the development and validation cohorts. In addition, other cell types shared a similar distribution to that of the actual classification (Figures 6(b) and 6(c)). Since the DEG analysis suggested that an aberrant cell cycle contributes to the TACE nonresponder status and CDK inhibitors are candidate therapeutic agents, we evaluated whether tumor stemness differs between responders and nonresponders. We calculated the mRNAsi for each sample using the OCLR method. As shown in Figure 6(d), the TACE nonresponse group showed higher mRNAsi values than those of the response group. We later compared the mRNAsi between the predicted response and nonresponse groups in the development and external validation cohorts and found that our model could discern those with higher mRNAsi values in both cohorts (Figures 6(e) and 6(f)), providing insight into factors contributing to the predictive value of our gene signature.

## 4. Discussion

With the recent emphasis on precision medicine and the rapid decline in genomic profiling costs, the use of accumulating data to develop novel approaches to guide disease diagnosis and treatment has become a standard approach. In this study, we investigated differential gene expression patterns between TACE responders and nonresponders and developed a TACE-specific SVM-based model using 10 genes. We successfully validated the efficacy of the model for predicting outcomes after TACE.

Most of the target genes were not associated with TACE and only a few have been studied in HCC. TSPAN8, S100A8, TYRO3, LOX, and PEG10, which were upregulated in nonresponders in our study, have been identified as indicators of a poor prognosis in HCC and could promote HCC progression by multiple mechanisms, such as proliferation, invasion, and metastasis [16–18]. Additionally, the overexpression of TYRO3 mediates sorafenib resistance and could serve as a potential target of cabozantinib [19, 20]. LOX, as an extracellular matrix (ECM) remodeling enzyme, might stiffen the ECM and support angiogenesis surrounding the tumor tissue, thereby contributing to the TACE nonresponse phenotype [21]. The remaining five genes were downregulated in TACE nonresponders, including SPARCL1, AQP1, TIAM1, HERC6, and FABP4. The functions of these five genes were not as clear as those of their upregulated counterparts. TIAM1 and FABP4 have been found to promote HCC progression by promoting metastasis and tumorigenesis [22, 23]. AQP1, which is mostly expressed in the membrane of microvessels, could indicate the extent of neovascularization or angiogenesis of the tumor, and higher AQP1 expression in HCC usually indicates a worse prognosis [24]. SPARCL1 has the opposite effect on angiogenesis. SPARCL1, also known as Hevin, works together with SPARC to diminish angiogenesis HCC and delay in vivo tumor growth [25]. The functions of these dysregulated genes and particularly their impact on the development of HCC and the response to TACE require further research.

One of the main differences between TACE and traditional chemotherapy is the additional embolization of the tumor-feeding artery; accordingly, many researchers have focused on pathways involved in hypoxia and angiogenesis to explore variation among individuals in the TACE response. Some studies have revealed a negative correlation between the pre-TACE levels of hypoxia-related biomarkers, such as vascular endothelial growth factor (VEGF) and hypoxia-induced factor 1*α* (HIF-1*α*) and survival outcomes [10, 26]. However, our GSVA result and PPI network failed to discern a direct pretherapy overactivated hypoxia-related biological process in TACE nonresponders. Instead, we found that pathways related to an aberrant cell cycle and proliferation, including G2/M checkpoint, E2F, MYC, and mTORC1 were significantly enriched in TACE nonresponders [27–29]. However, some previous studies have suggested that there is a positive correlation between hypoxia and activated mTORC1 and E2F pathways [30]. Additionally, an enrichment analysis of DEGs in our study recapitulated the relationship between the augmented IL-17 pathway and TACE nonresponse. IL-17 predicts a poor prognosis in HCC, in part due to its ability to promote angiogenesis [31]. Lower levels of IL-17 are favorable for the survival of patients treated with the combination of apatinib and TACE compared with TACE alone [32]. In our study, overactivation of the IL-17 pathway was observed in the nonresponse group; however, we found no obvious elevation in the expression levels of IL-17 family molecules. Further research is required to elucidate the role of IL-17 in the TACE response.

To explore the mechanisms underlying the predictive value of our model, we focused on differences in infiltrating immune cells among groups. With the advent of immunotherapy in HCC management, the famous immune-suppressing CD4+ CD25+ Foxp3+ regulatory T cells (Tregs) got increasing attention recently [33]. Previous study disclosed a negative correlation between pre-TACE Tregs fraction and survival after operation [34]. But no significant association between pre-TACE Tregs fraction and TACE response status was found which is consistent with our results [35]. TACE nonresponders had higher frequencies of macrophage M0 cells and lower frequencies of resting mast cells than those of responders. These characteristics were captured by our model and detected in external validation cohorts. M0 macrophages are commonly known as nonactivated macrophages and constitute tumor-associated macrophages, along with M1 and M2 phenotypes. The higher fraction of M0 in nonresponders could result from the increased recruitment of circulating monocytes. Altered tumor environments, such as hypoxia, inflammation, chemicals released by tumor cells, and augmented inflammation, could facilitate the accumulation of macrophages [36]. Although the impact of a large population of macrophages in HCC is controversial, most studies regard it as an indicator of a poorer prognosis [37]. In particular, S100A8 and TYRO3, which were predicted to increase the risk of nonresponse in our model, were associated with macrophage infiltration. Infiltrating macrophages can upregulate S100A8 expression in tumor cells and promote their invasion and migration [38]. TYRO3 could serve as a receptor on the surface of macrophages, mediating its interaction with tumor cells and potentiating its polarization toward the anti-inflammatory M2 phenotype [39].

The functional enrichment analyses of DEGs, PPI subnetwork, and CMap implied that cell cycle progression is significantly expediated in the nonresponse group. Accordingly, we predicted and demonstrated the high stemness feature of nonresponders in our model. A previous study has found that HCC with low expression levels of stemness-related markers, such as keratin 19 or epithelial cell adhesion molecule (EpCAM), could show better outcomes after TACE, such as fewer residual tumors and more complete tumor necrosis [40]. These results are consistent with ours and suggest that tumor stemness is a potential therapeutic target.

We believe that in the era of precision and personalized medicine, it is increasingly important to weaponize gene information from individual patients to find appropriate therapies. A gene signature was previously developed from GSE14520 alone to forecast patient responses to TACE; however, the primary grouping of the training cohort was retrospectively based on survival outcomes after TACE which is confounded by many factors, and the criteria for responses were different from those in common clinical practice [10]. Our model was developed using the clinical phenotype to effectively label the training cohort. However, the lack of related clinical information and diagnostic criteria also partially impaired the credibility of our results. Deeper integration with clinical information could improve our model.

## 5. Conclusion

Our model based on expression of 10 genes could potentially predict HCC patients' response and prognosis after TACE treatment. The discriminating power was TACE-specific.

## Figures and Tables

**Table 1:** Comparative performance between different predictive models using selective genes.

Models	AUC	F1 score	Accuracy	Youden index	Sensitivity	Specificity	PPV	NPV
SVM	0.930	0.798	0.823	0.643	0.808	0.835	0.793	0.851
ANN	0.922	0.816	0.843	0.690	0.815	0.865	0.832	0.867
Log	0.918	0.819	0.847	0.686	0.815	0.871	0.830	0.865
XGBoost	0.858	0.762	0.793	0.581	0.769	0.812	0.763	0.827
RF	0.839	0.726	0.760	0.515	0.738	0.776	0.737	0.804

Log: Lasso-logistic regression; SVM: support vector machine; ANN: artificial neural network; RF: random forest; XGBoost: eXtreme gradient boosting-based tree model.

**Table 2:** Top 20 genes and their corresponding coefficients in SVM model.

Gene	Occurrence	Ranking sum	Average rank	Coefficient
SLC9A3-AS1	10/10	31	3.1	
TSPAN8	10/10	31	3.1	0.053
SLAIN1	10/10	68	6.8	
SPARCL1	10/10	70	7	−0.098
PKIB	10/10	121	12.1	
S100A8	10/10	128	12.8	0.064
AQP1	10/10	212	21.2	−0.069
ERAP2	9/10	98	10.9	
TYRO3	9/10	135	15	0.084
TIAM1	9/10	171	19	−0.054
HERC6	9/10	224	24.9	−0.054
ZC3HAV1L	9/10	248	27.6	
LOX	9/10	306	34	0.065
FABP4	8/10	178	22.2	−0.033
PEG10	8/10	194	24.2	0.027
SLC35G1	8/10	256	32	
VWF	7/10	68	9.7	
CXCL11	7/10	89	12.7	
DHRS2	7/10	93	13.3	
ADH1C	7/10	103	14.7	

SLC9A3-AS1: SLC9A3 antisense RNA 1; TSPAN8: Tetraspanin 8; SLAIN1: SLAIN motif family member 1; SPARCL1: SPARC-like 1; PKIB: cAMP-dependent protein kinase inhibitor beta; S100A8: S100 calcium binding protein A8; AQP1: aquaporin 1; ERAP2: endoplasmic reticulum aminopeptidase 2; TYRO3: TYRO3 protein tyrosine kinase; TIAM1: TIAM Rac1 associated GEF 1; HERC6: HECT and RLD domain-containing E3 ubiquitin protein ligase family member 6; ZC3HAV1L: zinc finger CCCH-type containing, antiviral 1 like; LOX: lysyl oxidase; FABP4: fatty acid binding protein 4; PEG10: paternally expressed 10; SLC35G1: solute carrier family 35 member G1; VWF: von Willebrand factor; CXCL11: C-X-C motif chemokine ligand 11; DHRS2: dehydrogenase/reductase 2; ADH1C: alcohol dehydrogenase 1C.

**Table 3:** Cox regression for OS of patients receiving TACE treatment (*n* = 104).

Feature	Univariate analysis			Multivariate analysis		
	Hazard ratio	95% CI (HR)	*p* value	Hazard ratio	95% CI (HR)	*p* value
Gender (male VS female)	1.30	0.40–4.20	0.66			
Age (>VS ≤ 50)	1.07	0.59–1.94	0.83			
Predicted response (nonresponse VS response)	2.12	1.16–3.87	0.01^*∗*^	1.93	1.02–3.64	0.04^*∗*^
HBV (positive VS negative)	0.75	0.18–3.10	0.69			
AFP (>VS ≤ 300 ng/ml)	1.35	0.74–2.45	0.33			
ALT (>VS ≤ 50 U/L)	0.85	0.46–1.55	0.59			
Cirrhosis (presence VS absence)	7.15	0.98–51.99	0.05			
Main tumor size (>VS ≤ 5 cm)	1.88	1.03–3.42	0.04^*∗*^	1.40	0.67–2.93	0.38
Multinodular (presence VS absence)	1.17	0.58–2.38	0.66			
BCLC stage (B and C VS 0 and A)	2.76	1.45–5.22	<0.005	1.98	0.93–4.24	0.08

HBV, hepatitis B virus; AFP, alpha fetoprotein; ALT, alanine aminotransferase; BCLC stage, Barcelona clinic liver cancer staging (there were no BCLC-D patients included in the research) (^*∗*^*p* < 0.05).

## Data Availability

The transcriptome used in this study is available in GEO database (https://www.ncbi.nlm.nih.gov/geo/) under accession numbers GSE14520 and GSE104580.

## Supplementary Materials

Supplementary Materials
*Supplementary Figure 1.* Survival prediction efficacy of our model. A, the 1-, 3-, and 5-year time-dependent ROC curve as well as relative AUC assessing the efficacy of our model in predicting OS of patients receiving TACE. B and C, the 1-, 3-, and 5-year time-dependent ROC curve as well as relative AUC assessing the efficacy of our model in predicting OS and RFS of patients receiving adjuvant TACE. D, the 1-, 3-, and 5-year time-dependent ROC curve as well as relative AUC assessing the efficacy of our model in predicting OS of patients receiving postrecurrence TACE. E and F, the 1-, 3-, and 5-year time-dependent ROC curve as well as relative AUC assessing the efficacy of our model in predicting OS and RFS of patients receiving resection only. G, calculated AUC value at any given time points between 10 and 60 months in different patient groups. *Supplementary Table 1.* DEGs between TACE responders and nonresponders. *Supplementary Table 2.* More effective drugs in TACE responders. *Supplementary Table 3.* More effective drugs in TACE nonresponders. *Supplementary Table 4.* Performance of five models based on 373 DEGs. *Supplementary Table 5.* Top 20 important genes of each model.
