# Supplementary material for: A 10-Gene Signature Identified by Machine Learning for Predicting the Response to Transarterial Chemoembolization in Patients with Hepatocellular Carcinoma
Source: J Oncol. 2022 Jan 24;2022:3822773. doi: 10.1155/2022/3822773 (PMC8803430; doi:10.1155/2022/3822773)
Supplement: Supplementary Materials — Supplementary Figure 1. Survival prediction efficacy of our model. A, the 1-, 3-, and 5-year time-dependent ROC curve as well as relative AUC assessing the efficacy of our model in predicting OS of patients receiving TACE. B and C, the 1-, 3-, and 5-year time-dependent ROC curve as well as relative AUC assessing the efficacy of our model in predicting OS and RFS of patients receiving adjuvant TACE. D, the 1-, 3-, and 5-year time-dependent ROC curve as well as relative AUC assessing the efficacy of our model in predicting OS of patients receiving postrecurrence TACE. E and F, the 1-, 3-, and 5-year time-dependent ROC curve as well as relative AUC assessing the efficacy of our model in predicting OS and RFS of patients receiving resection only. G, calculated AUC value at any given time points between 10 and 60 months in different patient groups. Supplementary Table 1. DEGs between TACE responders and nonresponders. Supplementary Table 2. More effective drugs in TACE responders. Supplementary Table 3. More effective drugs in TACE nonresponders. Supplementary Table 4. Performance of five models based on 373 DEGs. Supplementary Table 5. Top 20 important genes of each model. [file 3822773.f1.zip › 3822773.f1/supplemental tables.docx]

**Supplementary Table 1.** DEGs between TACE responders and non-responders.

| logFC | AveExpr | t | P.Value | adj.P.Val | B | SYMBOL |
| --- | --- | --- | --- | --- | --- | --- |
| -1.0236 | 10.97088 | -3.27147 | 0.001335 | 0.013005 | -1.29534 | A1BG |
| -1.14338 | 6.097721 | -3.23575 | 0.001501 | 0.014131 | -1.4008 | NAT2 |
| 1.298341 | 12.57095 | 4.791851 | 4.02E-06 | 0.000204 | 4.048152 | CD24 |
| 1.339194 | 5.943512 | 5.538356 | 1.38E-07 | 2.12E-05 | 7.207466 | TROAP |
| -1.12499 | 4.711647 | -4.08788 | 7.16E-05 | 0.001503 | 1.375515 | SLC9A3-AS1 |
| -1.32807 | 8.286205 | -4.14055 | 5.84E-05 | 0.001308 | 1.564244 | SLC17A4 |
| 1.386534 | 4.695855 | 5.264433 | 4.93E-07 | 4.98E-05 | 6.012372 | MIR210HG |
| -1.02574 | 4.918497 | -3.19922 | 0.00169 | 0.015343 | -1.50758 | LINC01093 |
| -2.66488 | 7.092158 | -5.66756 | 7.48E-08 | 1.36E-05 | 7.784655 | LINC00844 |
| 1.133843 | 3.9242 | 3.989045 | 0.000105 | 0.001958 | 1.026599 | CDH10 |
| -1.12845 | 10.62598 | -4.88547 | 2.68E-06 | 0.000154 | 4.426987 | CYP3A7-CYP3A51P |
| -1.34842 | 7.979411 | -5.35199 | 3.30E-07 | 3.75E-05 | 6.390034 | AASS |
| -1.88882 | 7.890319 | -3.43776 | 0.000764 | 0.008614 | -0.79147 | DHRS2 |
| -1.01256 | 12.72855 | -6.02034 | 1.34E-08 | 4.54E-06 | 9.402096 | RIDA |
| -2.71001 | 6.87599 | -5.74445 | 5.17E-08 | 1.10E-05 | 8.132091 | GLYAT |
| -1.27321 | 8.800611 | -3.22646 | 0.001547 | 0.01444 | -1.42804 | ABCA8 |
| 1.667805 | 11.20063 | 7.637119 | 2.68E-12 | 1.83E-08 | 17.46314 | NDRG1 |
| 1.124609 | 8.733573 | 3.319295 | 0.00114 | 0.011575 | -1.15262 | CLGN |
| 2.609108 | 4.960528 | 4.772152 | 4.38E-06 | 0.000218 | 3.969111 | AGR2 |
| -1.22674 | 9.577019 | -4.34035 | 2.64E-05 | 0.000744 | 2.297072 | SLCO1B1 |
| 1.078298 | 5.631463 | 4.191195 | 4.79E-05 | 0.00112 | 1.747453 | CENPE |
| 1.057061 | 8.038573 | 3.964671 | 0.000115 | 0.002087 | 0.941589 | CENPF |
| 1.171636 | 2.928544 | 5.672897 | 7.29E-08 | 1.33E-05 | 7.808678 | IGF2BP1 |
| 2.317746 | 8.740709 | 5.920233 | 2.20E-08 | 6.35E-06 | 8.937144 | IGF2BP3 |
| 1.652628 | 8.898538 | 5.808229 | 3.80E-08 | 8.90E-06 | 8.422484 | IGF2BP2 |
| -1.25799 | 4.478955 | -4.38056 | 2.25E-05 | 0.000661 | 2.447722 | ADCY1 |
| -1.32941 | 7.398245 | -3.89221 | 0.000151 | 0.002568 | 0.691322 | MASP2 |
| 1.193698 | 3.628268 | 4.756977 | 4.67E-06 | 0.000228 | 3.908378 | GJB6 |
| -1.38148 | 8.550715 | -3.59531 | 0.000442 | 0.005822 | -0.29466 | ALDH1L1 |
| -1.6866 | 10.59446 | -4.53706 | 1.18E-05 | 0.00042 | 3.044168 | CFHR4 |
| -1.68135 | 8.791341 | -4.38869 | 2.17E-05 | 0.000643 | 2.478334 | CFHR3 |
| -1.25379 | 6.620062 | -3.36766 | 0.000969 | 0.010231 | -1.00647 | ADIRF |
| -1.8886 | 8.852295 | -5.45893 | 2.01E-07 | 2.74E-05 | 6.85687 | SLC27A5 |
| 1.264466 | 6.642144 | 4.545065 | 1.14E-05 | 0.000412 | 3.075093 | KIF2C |
| 1.035174 | 9.61488 | 4.498744 | 1.39E-05 | 0.00047 | 2.896688 | UBE2C |
| -1.04591 | 8.183625 | -6.68333 | 4.60E-10 | 5.51E-07 | 12.59101 | NUDT6 |
| -1.61445 | 8.065773 | -4.77345 | 4.35E-06 | 0.000218 | 3.974307 | ANXA10 |
| 1.64758 | 5.989773 | 5.361606 | 3.15E-07 | 3.65E-05 | 6.431771 | EGLN3 |
| 1.251052 | 3.392137 | 4.585481 | 9.66E-06 | 0.00037 | 3.23186 | SLC6A14 |
| -1.14026 | 7.933558 | -2.79517 | 0.005885 | 0.037557 | -2.6178 | PGLYRP2 |
| 1.353237 | 10.08182 | 3.363067 | 0.000984 | 0.01035 | -1.02043 | CTHRC1 |
| -1.15463 | 5.491808 | -2.84945 | 0.005013 | 0.033626 | -2.47634 | ACSM1 |
| -1.01186 | 6.414249 | -4.66624 | 6.88E-06 | 0.000297 | 3.548175 | RBP7 |
| -1.59443 | 8.590058 | -5.27699 | 4.66E-07 | 4.77E-05 | 6.066285 | APOA5 |
| 1.049261 | 4.937656 | 3.32311 | 0.001125 | 0.011457 | -1.14116 | ERP27 |
| 1.299082 | 6.242194 | 4.591728 | 9.41E-06 | 0.000364 | 3.256185 | SLAIN1 |
| -1.50113 | 11.67979 | -6.09666 | 9.20E-09 | 3.70E-06 | 9.759652 | ACSM2A |
| -1.78395 | 11.65769 | -5.33103 | 3.63E-07 | 3.96E-05 | 6.299257 | ADH1A |
| -2.02457 | 12.34803 | -5.74923 | 5.05E-08 | 1.09E-05 | 8.153781 | ADH1B |
| -2.17592 | 11.70804 | -6.67355 | 4.84E-10 | 5.51E-07 | 12.54269 | ADH1C |
| -2.16988 | 10.31338 | -4.1654 | 5.30E-05 | 0.001216 | 1.653899 | ADH4 |
| -1.22481 | 10.45227 | -4.68342 | 6.39E-06 | 0.000284 | 3.615998 | ADH6 |
| 1.999795 | 6.515336 | 4.300791 | 3.10E-05 | 0.000824 | 2.149846 | TRIM71 |
| -1.06108 | 4.641119 | -4.62143 | 8.31E-06 | 0.000339 | 3.37217 | SPATA18 |
| 1.14917 | 8.951363 | 5.2164 | 6.14E-07 | 5.85E-05 | 5.806956 | ADM |
| -1.65175 | 7.27358 | -4.89376 | 2.59E-06 | 0.00015 | 4.460798 | ACOT12 |
| -1.67571 | 6.267505 | -6.0768 | 1.02E-08 | 3.82E-06 | 9.666358 | ADHFE1 |
| -1.11427 | 3.644815 | -3.07269 | 0.002532 | 0.020604 | -1.86937 | TTC36 |
| -1.05859 | 10.502 | -3.53162 | 0.000553 | 0.006846 | -0.49775 | HJV |
| 1.074362 | 4.898771 | 3.669015 | 0.000341 | 0.004798 | -0.05589 | CTNND2 |
| -1.01341 | 8.602239 | -6.37862 | 2.22E-09 | 1.24E-06 | 11.10271 | CTSO |
| -1.19104 | 4.756228 | -4.26177 | 3.62E-05 | 0.000914 | 2.005669 | MGC32805 |
| -1.34531 | 6.055287 | -7.52225 | 5.06E-12 | 2.59E-08 | 16.86118 | BTNL9 |
| -1.38314 | 3.936501 | -3.85416 | 0.000173 | 0.002858 | 0.561423 | CYP1A1 |
| -1.69536 | 9.886567 | -4.60596 | 8.87E-06 | 0.000349 | 3.311689 | CYP1A2 |
| -1.22586 | 7.729982 | -4.49428 | 1.41E-05 | 0.000478 | 2.879568 | RUNDC3B |
| -1.825 | 8.015585 | -4.61772 | 8.44E-06 | 0.000342 | 3.357649 | CYP2A6 |
| -1.2491 | 7.142716 | -4.28628 | 3.28E-05 | 0.000852 | 2.096091 | CYP2A7 |
| -1.85584 | 12.28639 | -5.71073 | 6.08E-08 | 1.19E-05 | 7.979366 | CYP2C8 |
| -1.74506 | 11.70776 | -5.31001 | 4.00E-07 | 4.23E-05 | 6.208449 | CYP2C9 |
| -1.00919 | 8.897537 | -3.76797 | 0.000238 | 0.003647 | 0.270952 | CYP2C18 |
| 1.065895 | 6.23232 | 4.816853 | 3.61E-06 | 0.00019 | 4.148811 | CDCA2 |
| -1.98173 | 10.48956 | -5.39005 | 2.76E-07 | 3.42E-05 | 6.555493 | CYP3A4 |
| -1.5538 | 8.043019 | -4.12762 | 6.14E-05 | 0.001343 | 1.517714 | CYP4A11 |
| -2.33144 | 7.648741 | -4.54192 | 1.16E-05 | 0.000414 | 3.062921 | CYP7A1 |
| -1.90165 | 8.7531 | -3.78338 | 0.000225 | 0.003494 | 0.32249 | CYP8B1 |
| 1.176245 | 6.009351 | 4.477821 | 1.51E-05 | 0.000504 | 2.816552 | SLC35G1 |
| -1.20166 | 7.36829 | -4.23517 | 4.02E-05 | 0.000987 | 1.907948 | DAO |
| -1.029 | 7.756715 | -3.93674 | 0.000127 | 0.002266 | 0.844693 | TMEM30B |
| -1.28209 | 6.884797 | -4.59812 | 9.16E-06 | 0.000357 | 3.281082 | NAGS |
| 1.020069 | 5.382981 | 6.381089 | 2.19E-09 | 1.24E-06 | 11.11462 | C18orf54 |
| 1.486887 | 4.203745 | 4.22296 | 4.22E-05 | 0.00102 | 1.863252 | KANK4 |
| -1.09309 | 7.425995 | -3.3258 | 0.001115 | 0.011372 | -1.13307 | SYNPO2 |
| -1.71625 | 9.238336 | -5.38619 | 2.81E-07 | 3.42E-05 | 6.538676 | AFM |
| 2.271239 | 8.643047 | 3.795156 | 0.000216 | 0.00336 | 0.361995 | AFP |
| -1.42745 | 8.295504 | -3.99282 | 0.000103 | 0.001942 | 1.03981 | DNASE1L3 |
| -1.14333 | 12.82485 | -5.58542 | 1.11E-07 | 1.87E-05 | 7.416746 | ABAT |
| -1.16011 | 5.574322 | -3.61905 | 0.000407 | 0.005467 | -0.2182 | DPT |
| 1.347621 | 5.207307 | 3.243023 | 0.001466 | 0.013882 | -1.3794 | SLC26A3 |
| 1.0756 | 7.126214 | 4.940007 | 2.11E-06 | 0.000128 | 4.650067 | ARID3A |
| 1.011503 | 10.26867 | 4.713024 | 5.64E-06 | 0.00026 | 3.73327 | DSG2 |
| 1.671538 | 4.851092 | 4.717566 | 5.53E-06 | 0.000257 | 3.751308 | DUSP9 |
| -1.20743 | 11.03584 | -4.0662 | 7.78E-05 | 0.001593 | 1.298415 | AGXT |
| -1.14447 | 4.436792 | -6.72909 | 3.62E-10 | 5.51E-07 | 12.81765 | VSTM4 |
| -1.1615 | 4.858928 | -5.72071 | 5.80E-08 | 1.18E-05 | 8.024514 | LDHD |
| 1.625965 | 3.46724 | 5.825109 | 3.50E-08 | 8.75E-06 | 8.499667 | ENO2 |
| -1.1871 | 7.860493 | -4.15128 | 5.60E-05 | 0.001265 | 1.602884 | EPHX2 |
| 1.274807 | 3.99812 | 4.691405 | 6.18E-06 | 0.000279 | 3.64757 | EPO |
| 1.126882 | 4.650084 | 3.333967 | 0.001085 | 0.011154 | -1.10847 | EREG |
| -1.34019 | 4.160311 | -4.38954 | 2.17E-05 | 0.000641 | 2.481524 | ESR1 |
| 1.249181 | 7.857937 | 4.952898 | 2.00E-06 | 0.000123 | 4.703051 | ETV1 |
| -1.42569 | 11.84588 | -3.60269 | 0.000431 | 0.005701 | -0.27095 | F9 |
| -1.52176 | 7.565517 | -5.71708 | 5.90E-08 | 1.18E-05 | 8.008066 | F11 |
| -1.20956 | 10.44217 | -4.37814 | 2.27E-05 | 0.000666 | 2.438628 | F12 |
| -1.07121 | 8.097717 | -2.98584 | 0.003317 | 0.025017 | -2.11033 | FABP4 |
| -1.0512 | 2.791287 | -3.30242 | 0.001205 | 0.012051 | -1.20319 | ALDH3A1 |
| 1.310664 | 3.62398 | 4.445147 | 1.73E-05 | 0.000541 | 2.691969 | RFX6 |
| 1.431444 | 8.213879 | 4.127353 | 6.14E-05 | 0.001343 | 1.51677 | FKBP1B |
| 2.036019 | 7.191249 | 3.595725 | 0.000442 | 0.005821 | -0.29333 | DKK1 |
| 1.016879 | 7.486855 | 4.445213 | 1.73E-05 | 0.000541 | 2.692222 | TPX2 |
| -1.1348 | 8.439997 | -4.59486 | 9.29E-06 | 0.00036 | 3.268386 | AKR7A3 |
| 1.357282 | 6.315822 | 4.324955 | 2.81E-05 | 0.000771 | 2.239644 | TTC39A |
| -1.09063 | 5.595381 | -3.7671 | 0.000239 | 0.003655 | 0.268064 | EXPH5 |
| 1.490369 | 10.27859 | 2.814557 | 0.005559 | 0.036048 | -2.56755 | PEG10 |
| 1.184939 | 7.852111 | 5.814312 | 3.69E-08 | 8.86E-06 | 8.450282 | HIC2 |
| 1.294767 | 6.349759 | 2.97887 | 0.003389 | 0.025385 | -2.12939 | FMO1 |
| -1.09585 | 11.9139 | -3.61487 | 0.000413 | 0.005532 | -0.23168 | FMO3 |
| 1.107944 | 4.42042 | 5.335841 | 3.55E-07 | 3.92E-05 | 6.320075 | TBC1D30 |
| -1.08228 | 9.016217 | -3.91901 | 0.000136 | 0.002373 | 0.783453 | ABCA6 |
| -1.35548 | 5.80775 | -4.80963 | 3.73E-06 | 0.000193 | 4.119688 | SEC14L2 |
| 1.112727 | 4.080964 | 3.816045 | 0.0002 | 0.003189 | 0.43231 | LPAR3 |
| 1.050923 | 6.571724 | 3.018754 | 0.002996 | 0.023307 | -2.01972 | SLC7A11 |
| 1.056821 | 7.16124 | 3.879214 | 0.000158 | 0.002658 | 0.646849 | KIF4A |
| -1.57191 | 4.854995 | -4.50257 | 1.36E-05 | 0.000467 | 2.911358 | FNDC5 |
| -1.48637 | 5.745992 | -4.81777 | 3.60E-06 | 0.000189 | 4.152501 | LINC01018 |
| 1.172392 | 3.662743 | 4.181705 | 4.97E-05 | 0.001155 | 1.712987 | GABRA2 |
| 1.135415 | 4.395363 | 3.062757 | 0.002612 | 0.021096 | -1.89725 | MAP7D2 |
| 1.457346 | 7.735829 | 5.403194 | 2.60E-07 | 3.33E-05 | 6.612813 | QPCT |
| 1.256863 | 9.544792 | 5.324346 | 3.75E-07 | 4.04E-05 | 6.270357 | BAMBI |
| -1.16148 | 6.842706 | -5.58209 | 1.12E-07 | 1.89E-05 | 7.401897 | FAM149A |
| -1.19861 | 8.206555 | -3.29626 | 0.00123 | 0.012228 | -1.22159 | MYRIP |
| 1.371934 | 6.228908 | 4.614332 | 8.56E-06 | 0.000344 | 3.344397 | STEAP2 |
| 1.128391 | 9.305595 | 3.778705 | 0.000229 | 0.003535 | 0.30684 | PCOLCE2 |
| -1.92215 | 7.192535 | -4.52563 | 1.24E-05 | 0.000434 | 3.000067 | FETUB |
| 1.126734 | 11.85656 | 5.349871 | 3.33E-07 | 3.77E-05 | 6.380857 | TSPAN13 |
| -1.92827 | 5.997013 | -4.21972 | 4.28E-05 | 0.00103 | 1.851429 | GLS2 |
| -2.57478 | 8.096405 | -6.62802 | 6.14E-10 | 6.28E-07 | 12.31814 | GNMT |
| 1.024038 | 7.96999 | 3.673708 | 0.000335 | 0.004728 | -0.04055 | TNFRSF21 |
| 1.399627 | 3.686903 | 3.631032 | 0.00039 | 0.005299 | -0.17944 | TINAG |
| -1.13468 | 11.6318 | -3.88515 | 0.000155 | 0.002613 | 0.667152 | ANGPTL3 |
| -1.60512 | 9.169541 | -4.60071 | 9.06E-06 | 0.000354 | 3.291192 | GPLD1 |
| 1.047134 | 6.902458 | 2.907084 | 0.004218 | 0.029743 | -2.32353 | ADGRD1 |
| 1.219348 | 10.04133 | 7.035346 | 7.14E-11 | 2.08E-07 | 14.35401 | GAS2L3 |
| -1.06417 | 4.56039 | -3.56108 | 0.000499 | 0.006373 | -0.40418 | SLC25A47 |
| 1.205682 | 9.524692 | 4.556294 | 1.09E-05 | 0.000401 | 3.118542 | GSTM3 |
| 1.414068 | 7.805941 | 6.511586 | 1.12E-09 | 8.21E-07 | 11.74763 | HILPDA |
| -1.28171 | 9.133625 | -4.75723 | 4.67E-06 | 0.000228 | 3.909383 | DMGDH |
| 1.122526 | 4.653618 | 4.650952 | 7.34E-06 | 0.000309 | 3.487988 | LRP12 |
| -1.73106 | 7.248959 | -4.25515 | 3.72E-05 | 0.000934 | 1.981285 | GYS2 |
| -1.19136 | 5.823076 | -4.60886 | 8.76E-06 | 0.000347 | 3.323004 | HGFAC |
| 2.316291 | 7.501169 | 6.45287 | 1.52E-09 | 1.04E-06 | 11.46195 | HK2 |
| -2.47923 | 9.957881 | -5.47966 | 1.82E-07 | 2.59E-05 | 6.948058 | HPD |
| -1.09564 | 11.1402 | -3.25268 | 0.00142 | 0.013598 | -1.35095 | HPR |
| -1.05238 | 12.1848 | -3.66497 | 0.000345 | 0.004844 | -0.06909 | HPX |
| -1.57729 | 13.63036 | -4.9779 | 1.79E-06 | 0.000115 | 4.806072 | HRG |
| -1.76097 | 10.63032 | -4.36454 | 2.40E-05 | 0.000691 | 2.387565 | HSD11B1 |
| 1.351168 | 7.945872 | 4.499302 | 1.38E-05 | 0.000469 | 2.898827 | BIRC5 |
| -1.0576 | 4.898007 | -3.80077 | 0.000211 | 0.003313 | 0.380847 | HTR2B |
| -1.21042 | 10.19377 | -3.53588 | 0.000545 | 0.006782 | -0.48427 | IFI27 |
| -1.6431 | 8.983854 | -6.11667 | 8.33E-09 | 3.63E-06 | 9.853818 | IFIT1 |
| -1.00089 | 7.730817 | -3.04108 | 0.002795 | 0.02216 | -1.95777 | IGF1 |
| -1.74823 | 5.903766 | -5.8127 | 3.72E-08 | 8.86E-06 | 8.442897 | ACSM2B |
| -1.13499 | 10.10941 | -3.38365 | 0.000919 | 0.009887 | -0.95777 | IGFBP2 |
| 1.013612 | 3.766488 | 5.123112 | 9.36E-07 | 7.46E-05 | 5.411648 | IGSF1 |
| -1.48601 | 7.971616 | -6.5596 | 8.75E-10 | 7.47E-07 | 11.98225 | FAS |
| 1.393863 | 9.178185 | 3.911567 | 0.00014 | 0.002423 | 0.757825 | CXCL8 |
| -1.38802 | 7.134434 | -5.47553 | 1.86E-07 | 2.60E-05 | 6.929875 | AQP1 |
| -1.52096 | 10.11509 | -4.41813 | 1.93E-05 | 0.000587 | 2.589458 | AQP9 |
| -1.41631 | 8.128658 | -4.36206 | 2.42E-05 | 0.000696 | 2.378275 | IRF6 |
| -1.36069 | 8.462961 | -3.89114 | 0.000151 | 0.002574 | 0.687661 | AR |
| 1.079593 | 7.452894 | 4.134334 | 5.98E-05 | 0.001322 | 1.541861 | ITGA2 |
| -1.21283 | 11.61691 | -4.37904 | 2.26E-05 | 0.000664 | 2.442009 | ITIH4 |
| 1.083635 | 4.819142 | 3.985027 | 0.000106 | 0.001975 | 1.012556 | NCR3LG1 |
| -1.13226 | 8.174737 | -4.45934 | 1.63E-05 | 0.000527 | 2.74601 | KCNJ8 |
| -1.06419 | 8.951156 | -4.59622 | 9.23E-06 | 0.000359 | 3.273686 | KLKB1 |
| 1.865725 | 5.533185 | 5.571054 | 1.18E-07 | 1.94E-05 | 7.352735 | ARG2 |
| -1.79855 | 6.042295 | -6.07312 | 1.03E-08 | 3.82E-06 | 9.6491 | SLC22A25 |
| 1.129906 | 9.651601 | 3.693729 | 0.000312 | 0.00448 | 0.025071 | C12orf75 |
| -1.78135 | 9.214453 | -4.73738 | 5.08E-06 | 0.000242 | 3.830143 | C3P1 |
| 1.411971 | 9.197512 | 4.924643 | 2.26E-06 | 0.000136 | 4.587043 | TRNP1 |
| 2.673709 | 4.403785 | 5.980838 | 1.63E-08 | 5.15E-06 | 9.218081 | LIN28B |
| -1.63315 | 10.75888 | -3.5593 | 0.000502 | 0.006393 | -0.40984 | LECT2 |
| -1.25202 | 5.822327 | -3.97884 | 0.000109 | 0.002011 | 0.990967 | C3orf85 |
| 1.412964 | 7.590611 | 5.027954 | 1.43E-06 | 0.0001 | 5.01346 | LOX |
| 1.246829 | 4.306212 | 4.210973 | 4.43E-05 | 0.001056 | 1.819473 | LRP4 |
| 1.752251 | 9.507164 | 3.24304 | 0.001466 | 0.013882 | -1.37935 | EPCAM |
| 2.08312 | 6.958979 | 5.380559 | 2.89E-07 | 3.44E-05 | 6.514166 | MEP1A |
| 1.098124 | 4.034107 | 3.961656 | 0.000116 | 0.002099 | 0.931104 | MLF1 |
| 1.593268 | 5.311192 | 5.164259 | 7.78E-07 | 6.78E-05 | 5.585411 | MMP1 |
| 1.483706 | 7.238797 | 5.059508 | 1.24E-06 | 9.04E-05 | 5.144929 | MMP9 |
| 3.667588 | 7.129318 | 7.408178 | 9.47E-12 | 3.88E-08 | 16.26721 | MMP12 |
| 1.491712 | 7.673985 | 3.758419 | 0.000247 | 0.003739 | 0.239098 | ASNS |
| -1.08954 | 9.178386 | -6.29121 | 3.46E-09 | 1.82E-06 | 10.68273 | TMEM150C |
| 1.114651 | 5.514183 | 6.63706 | 5.85E-10 | 6.28E-07 | 12.36267 | MYBL2 |
| -1.12105 | 12.8426 | -3.88784 | 0.000153 | 0.002595 | 0.676359 | SERPINC1 |
| 1.047979 | 8.98341 | 4.10523 | 6.69E-05 | 0.001431 | 1.437482 | NEK2 |
| 1.268855 | 6.154402 | 3.098317 | 0.002335 | 0.019477 | -1.79713 | NRCAM |
| 2.239939 | 7.375441 | 3.784809 | 0.000224 | 0.003478 | 0.327285 | NTS |
| -1.12598 | 5.433105 | -3.64457 | 0.000372 | 0.005123 | -0.13551 | OGN |
| -1.4202 | 9.49975 | -3.44379 | 0.000749 | 0.00849 | -0.77281 | OTC |
| 1.177994 | 9.014664 | 3.412343 | 0.000834 | 0.009185 | -0.86986 | DUXAP10 |
| 1.109766 | 7.233998 | 5.256671 | 5.11E-07 | 5.13E-05 | 5.979091 | PAFAH1B3 |
| 1.507586 | 7.582799 | 4.445802 | 1.72E-05 | 0.000541 | 2.694461 | SERPINE1 |
| 1.167071 | 5.612634 | 3.257979 | 0.001396 | 0.01348 | -1.33529 | MYEF2 |
| -1.01319 | 4.245231 | -3.50536 | 0.000606 | 0.007284 | -0.58058 | PCDH7 |
| -1.43014 | 10.88835 | -3.16487 | 0.001888 | 0.01664 | -1.60706 | PCK1 |
| -1.10048 | 10.39427 | -6.07447 | 1.03E-08 | 3.82E-06 | 9.655431 | PCK2 |
| -1.50655 | 6.063986 | -3.3323 | 0.001091 | 0.011195 | -1.11348 | HAO2 |
| -1.04912 | 11.39961 | -4.42786 | 1.85E-05 | 0.000571 | 2.626343 | DCXR |
| -1.23161 | 11.5019 | -5.60679 | 9.99E-08 | 1.72E-05 | 7.512121 | PIPOX |
| 1.024313 | 9.550329 | 4.522956 | 1.25E-05 | 0.000436 | 2.98977 | SNX7 |
| 1.186381 | 6.60046 | 3.006363 | 0.003114 | 0.023898 | -2.05393 | GULP1 |
| 1.017497 | 4.119609 | 3.58952 | 0.000452 | 0.005908 | -0.31325 | PDE9A |
| 1.065802 | 4.948865 | 4.019418 | 9.31E-05 | 0.001805 | 1.133107 | ENPP3 |
| -1.61672 | 9.778231 | -4.61527 | 8.53E-06 | 0.000344 | 3.34806 | UPB1 |
| 1.018091 | 8.616662 | 4.13214 | 6.03E-05 | 0.001329 | 1.533973 | STK26 |
| -1.80834 | 5.52232 | -5.40541 | 2.57E-07 | 3.32E-05 | 6.622472 | PFKFB1 |
| 1.007327 | 5.751275 | 5.566652 | 1.21E-07 | 1.97E-05 | 7.333148 | PFKFB4 |
| 1.001438 | 7.737634 | 3.079117 | 0.002481 | 0.020313 | -1.85131 | PFN2 |
| -1.20098 | 9.019099 | -2.90347 | 0.004264 | 0.029937 | -2.3332 | PIGR |
| 1.087304 | 4.377237 | 5.125429 | 9.26E-07 | 7.44E-05 | 5.42141 | PKM |
| -1.23285 | 5.93526 | -5.46382 | 1.96E-07 | 2.70E-05 | 6.878364 | SEPTIN4 |
| 1.248108 | 3.474185 | 4.565187 | 1.05E-05 | 0.000391 | 3.153012 | TREM1 |
| -1.11148 | 10.87704 | -4.01655 | 9.42E-05 | 0.001818 | 1.123033 | HAO1 |
| -1.27497 | 11.70526 | -4.18685 | 4.87E-05 | 0.001137 | 1.731666 | PON1 |
| 1.085015 | 9.42771 | 4.456495 | 1.65E-05 | 0.000531 | 2.735161 | ANLN |
| -1.30317 | 11.66228 | -6.74335 | 3.36E-10 | 5.51E-07 | 12.88843 | PON3 |
| -1.11544 | 8.164172 | -4.30317 | 3.07E-05 | 0.000821 | 2.158679 | PALMD |
| -1.04511 | 5.45158 | -5.18154 | 7.19E-07 | 6.52E-05 | 5.658666 | PPL |
| -1.09159 | 8.957369 | -3.5237 | 0.000569 | 0.006982 | -0.52279 | ACSM5 |
| -1.02011 | 4.344793 | -4.28701 | 3.27E-05 | 0.000851 | 2.098797 | HERC6 |
| 1.016784 | 8.054493 | 2.839076 | 0.00517 | 0.034353 | -2.50357 | SOBP |
| -1.31581 | 7.686246 | -4.58315 | 9.75E-06 | 0.000373 | 3.222776 | ANO1 |
| 1.186026 | 7.997959 | 5.213253 | 6.23E-07 | 5.88E-05 | 5.793543 | FIGN |
| 1.040975 | 8.206889 | 4.640951 | 7.65E-06 | 0.000319 | 3.448688 | LRRC1 |
| -1.12143 | 4.442716 | -4.55633 | 1.09E-05 | 0.000401 | 3.118702 | TMEM100 |
| -1.34461 | 8.312246 | -3.4839 | 0.000653 | 0.007647 | -0.6479 | FGGY |
| 1.855745 | 6.724235 | 5.40065 | 2.63E-07 | 3.34E-05 | 6.601712 | LARP6 |
| 1.104329 | 6.480432 | 4.9123 | 2.38E-06 | 0.000141 | 4.536513 | HJURP |
| 1.64156 | 5.953363 | 4.392764 | 2.14E-05 | 0.000636 | 2.493676 | PPP1R9A |
| 1.317886 | 7.2324 | 4.684864 | 6.36E-06 | 0.000282 | 3.6217 | DEPDC1 |
| 1.407461 | 4.970572 | 4.562076 | 1.07E-05 | 0.000394 | 3.140951 | PKIA |
| 1.456696 | 7.373143 | 3.371066 | 0.000958 | 0.010167 | -0.99612 | PKIB |
| -2.17643 | 6.521625 | -6.90022 | 1.47E-10 | 2.86E-07 | 13.67202 | OGDHL |
| 1.164431 | 6.981186 | 3.023888 | 0.002949 | 0.023031 | -2.00551 | INAVA |
| 1.114689 | 8.177005 | 4.877851 | 2.77E-06 | 0.000158 | 4.395959 | DEPDC1B |
| 1.222685 | 6.420503 | 4.375557 | 2.29E-05 | 0.000669 | 2.428929 | MAPK13 |
| -1.16875 | 11.72408 | -4.53412 | 1.20E-05 | 0.000424 | 3.032815 | AZGP1 |
| 1.55406 | 4.502341 | 4.352517 | 2.52E-05 | 0.000715 | 2.342534 | CYP26B1 |
| 1.645993 | 7.263248 | 5.309702 | 4.01E-07 | 4.23E-05 | 6.207119 | ARFGEF3 |
| 1.071228 | 7.690188 | 4.679251 | 6.51E-06 | 0.000286 | 3.599517 | SPC25 |
| 1.572063 | 7.275244 | 5.44587 | 2.13E-07 | 2.87E-05 | 6.79954 | PPM1H |
| -1.11578 | 5.026178 | -4.31046 | 2.98E-05 | 0.000805 | 2.185728 | CYP4F11 |
| 1.210286 | 8.751588 | 5.010683 | 1.55E-06 | 0.000105 | 4.941744 | BCAT1 |
| 1.338845 | 3.012733 | 4.464378 | 1.60E-05 | 0.000519 | 2.765213 | RBP2 |
| 1.169267 | 7.149943 | 4.04261 | 8.52E-05 | 0.001703 | 1.214869 | BCL2A1 |
| 1.187458 | 8.122529 | 4.129716 | 6.09E-05 | 0.001337 | 1.525259 | RGS1 |
| 1.063256 | 9.157564 | 4.733833 | 5.16E-06 | 0.000244 | 3.816027 | RGS2 |
| -1.08389 | 8.279335 | -4.10335 | 6.74E-05 | 0.001435 | 1.430762 | RORC |
| -1.37221 | 9.110625 | -5.49385 | 1.70E-07 | 2.47E-05 | 7.010622 | BDH1 |
| 1.254221 | 9.221009 | 3.802829 | 0.00021 | 0.003304 | 0.387787 | S100A8 |
| 1.825272 | 8.938258 | 5.228378 | 5.81E-07 | 5.64E-05 | 5.858066 | S100A9 |
| 1.709386 | 8.660574 | 2.907662 | 0.00421 | 0.029731 | -2.32198 | S100P |
| -1.49818 | 11.35048 | -3.98529 | 0.000106 | 0.001975 | 1.013471 | BHMT |
| -1.24634 | 7.724977 | -3.21404 | 0.001611 | 0.014867 | -1.4644 | CCL16 |
| 1.540635 | 11.58768 | 4.314345 | 2.93E-05 | 0.000797 | 2.200167 | CCL20 |
| -1.02395 | 6.192308 | -2.86714 | 0.004755 | 0.032365 | -2.42974 | CCL21 |
| -1.04353 | 6.332452 | -2.76902 | 0.006352 | 0.039907 | -2.68507 | CXCL11 |
| 1.695606 | 6.983041 | 3.448209 | 0.000738 | 0.0084 | -0.7591 | CXCL5 |
| -1.4451 | 6.933029 | -5.35866 | 3.20E-07 | 3.68E-05 | 6.418997 | RTP4 |
| -1.13874 | 8.63076 | -3.84288 | 0.000181 | 0.002955 | 0.523115 | ERAP2 |
| 1.846965 | 4.061076 | 4.357684 | 2.46E-05 | 0.000707 | 2.36188 | POPDC3 |
| -1.04845 | 7.830266 | -2.98694 | 0.003306 | 0.024954 | -2.1073 | FAM110C |
| -1.45076 | 8.927864 | -4.20871 | 4.47E-05 | 0.00106 | 1.811212 | ETNPPL |
| 1.050345 | 4.124063 | 4.323401 | 2.83E-05 | 0.000774 | 2.233858 | SIX1 |
| -1.00951 | 8.812485 | -2.85062 | 0.004996 | 0.033542 | -2.47329 | SLC1A1 |
| -1.26905 | 7.697308 | -2.94177 | 0.003796 | 0.027614 | -2.23024 | SLC1A2 |
| 1.392599 | 7.257349 | 6.593583 | 7.34E-10 | 6.54E-07 | 12.14886 | SLC2A1 |
| -1.17808 | 11.26081 | -4.06019 | 7.97E-05 | 0.001623 | 1.277081 | SLC2A2 |
| 1.147171 | 7.299306 | 5.47163 | 1.89E-07 | 2.63E-05 | 6.912713 | SLC2A3 |
| 1.400342 | 4.697297 | 4.03236 | 8.86E-05 | 0.001748 | 1.178686 | SLC2A5 |
| -1.23875 | 7.531647 | -3.82752 | 0.000191 | 0.003088 | 0.471064 | WNK3 |
| -1.52246 | 9.942504 | -5.12731 | 9.18E-07 | 7.44E-05 | 5.429344 | SLC6A1 |
| -1.03201 | 8.75696 | -4.01976 | 9.30E-05 | 0.001804 | 1.134304 | ABCC6P1 |
| 1.827422 | 6.079634 | 4.661901 | 7.00E-06 | 0.000299 | 3.531079 | SLC6A8 |
| -1.31737 | 5.702971 | -3.6956 | 0.00031 | 0.004466 | 0.031224 | ZG16 |
| -2.1945 | 8.713603 | -5.10227 | 1.03E-06 | 7.98E-05 | 5.323993 | SLC10A1 |
| -2.31866 | 8.605109 | -4.94276 | 2.09E-06 | 0.000128 | 4.661366 | SLC22A1 |
| 1.678961 | 4.852616 | 5.108886 | 9.98E-07 | 7.77E-05 | 5.351794 | SPAG4 |
| 2.168187 | 12.15012 | 4.684818 | 6.36E-06 | 0.000282 | 3.621516 | SPINK1 |
| 1.436298 | 12.56423 | 4.269968 | 3.50E-05 | 0.000894 | 2.035867 | SPP1 |
| -1.0712 | 5.529326 | -4.2813 | 3.35E-05 | 0.000865 | 2.077684 | SRD5A2 |
| 1.971377 | 9.488536 | 4.960672 | 1.93E-06 | 0.000121 | 4.735051 | SULT1C2 |
| 1.239925 | 4.584317 | 4.4703 | 1.56E-05 | 0.000512 | 2.787814 | SYT1 |
| -1.9432 | 6.664889 | -5.23926 | 5.53E-07 | 5.50E-05 | 5.904565 | TAT |
| 1.209198 | 6.504826 | 4.825104 | 3.49E-06 | 0.000187 | 4.182114 | BUB1 |
| 1.03774 | 3.656651 | 3.274714 | 0.001321 | 0.012886 | -1.28572 | TFF1 |
| -1.81327 | 7.727651 | -3.23338 | 0.001513 | 0.014221 | -1.40774 | THRSP |
| -1.23573 | 5.842417 | -4.22737 | 4.15E-05 | 0.001009 | 1.879398 | TIAM1 |
| 1.116715 | 11.11388 | 3.104594 | 0.002289 | 0.019168 | -1.77936 | TSPAN8 |
| -1.11597 | 12.76132 | -4.2187 | 4.29E-05 | 0.001032 | 1.847691 | C4BPA |
| 1.205188 | 8.144128 | 5.155812 | 8.08E-07 | 6.95E-05 | 5.549663 | TTK |
| -1.16816 | 5.434241 | -4.45067 | 1.69E-05 | 0.000537 | 2.71299 | TTPA |
| -1.45414 | 10.59079 | -4.41028 | 1.99E-05 | 0.000601 | 2.559788 | C6 |
| 1.205009 | 6.30495 | 4.617063 | 8.46E-06 | 0.000343 | 3.355079 | TNFSF4 |
| -1.38867 | 9.368892 | -3.26825 | 0.001349 | 0.013123 | -1.3049 | C7 |
| 1.086413 | 3.454089 | 6.89146 | 1.54E-10 | 2.86E-07 | 13.62802 | TYRO3 |
| 1.342057 | 4.72568 | 2.864408 | 0.004794 | 0.032536 | -2.43696 | UCHL1 |
| -1.29927 | 12.44534 | -4.04688 | 8.38E-05 | 0.001682 | 1.229977 | UGT2B15 |
| 1.468736 | 7.781925 | 3.64087 | 0.000377 | 0.005163 | -0.14754 | VIL1 |
| -1.56914 | 7.856107 | -6.12047 | 8.17E-09 | 3.63E-06 | 9.871738 | VWF |
| -1.6419 | 9.235382 | -4.98437 | 1.74E-06 | 0.000114 | 4.832827 | XDH |
| 1.381946 | 6.43022 | 3.890547 | 0.000152 | 0.002578 | 0.685639 | ZIC2 |
| 1.753453 | 6.975687 | 5.414074 | 2.47E-07 | 3.21E-05 | 6.660325 | ZNF711 |
| -1.05628 | 10.0259 | -4.89988 | 2.52E-06 | 0.000147 | 4.485772 | CA2 |
| 1.298609 | 2.994978 | 4.254526 | 3.73E-05 | 0.000935 | 1.979001 | CA9 |
| 1.802011 | 7.216313 | 6.039614 | 1.22E-08 | 4.32E-06 | 9.492148 | CA12 |
| -1.25121 | 7.932874 | -3.29603 | 0.001231 | 0.012228 | -1.22227 | TRPM8 |
| 1.142953 | 3.560886 | 4.243522 | 3.89E-05 | 0.000964 | 1.938575 | EPS8L3 |
| -1.71604 | 7.736294 | -5.13026 | 9.06E-07 | 7.37E-05 | 5.441759 | STEAP4 |
| 1.075872 | 6.734906 | 5.210224 | 6.31E-07 | 5.91E-05 | 5.780636 | SHCBP1 |
| 1.435401 | 7.801708 | 4.475359 | 1.53E-05 | 0.000506 | 2.807139 | PLBD1 |
| 1.159469 | 7.845814 | 6.518989 | 1.08E-09 | 8.19E-07 | 11.78374 | LPCAT1 |
| -1.19565 | 8.056325 | -5.4135 | 2.48E-07 | 3.21E-05 | 6.657827 | CPED1 |
| 1.61032 | 6.647704 | 3.584955 | 0.000459 | 0.005965 | -0.32789 | FRAS1 |
| 1.171819 | 7.53597 | 4.567117 | 1.04E-05 | 0.000389 | 3.1605 | ASRGL1 |
| -1.14185 | 4.269832 | -4.49222 | 1.42E-05 | 0.000479 | 2.87167 | MOGAT2 |
| -1.02956 | 7.434713 | -5.09136 | 1.08E-06 | 8.22E-05 | 5.278194 | PDGFD |
| 1.151321 | 4.450765 | 4.33916 | 2.66E-05 | 0.000745 | 2.292611 | TET1 |
| -1.13172 | 8.667432 | -5.14901 | 8.33E-07 | 7.08E-05 | 5.520905 | APOL3 |
| 2.684297 | 4.646165 | 6.017638 | 1.36E-08 | 4.54E-06 | 9.389491 | HMGA2 |
| 1.516906 | 6.194034 | 5.172784 | 7.48E-07 | 6.69E-05 | 5.621534 | COLEC12 |
| -1.29387 | 9.643143 | -3.1044 | 0.00229 | 0.019168 | -1.77991 | CFHR5 |
| 1.000318 | 10.60607 | 4.136475 | 5.93E-05 | 0.001319 | 1.54956 | SLC38A1 |
| 1.071854 | 8.508625 | 4.025204 | 9.11E-05 | 0.001775 | 1.153472 | NUF2 |
| -1.96163 | 8.462819 | -4.54211 | 1.16E-05 | 0.000414 | 3.063667 | RTP3 |
| -1.54946 | 7.352862 | -5.0242 | 1.46E-06 | 0.0001 | 4.997875 | RBP5 |
| -1.10639 | 5.520782 | -3.39714 | 0.000878 | 0.009548 | -0.91653 | BCO2 |
| 1.478674 | 7.965993 | 4.492641 | 1.42E-05 | 0.000479 | 2.873285 | CDCA7 |
| -1.07682 | 4.848538 | -2.8861 | 0.004493 | 0.031068 | -2.37949 | FCAMR |
| -1.85412 | 10.13527 | -8.84389 | 2.76E-15 | 5.66E-11 | 23.98124 | SPARCL1 |
| 1.600717 | 7.962068 | 4.104009 | 6.73E-05 | 0.001433 | 1.433116 | C15orf48 |
| -1.30271 | 6.493421 | -5.70934 | 6.12E-08 | 1.19E-05 | 7.973088 | ZMAT1 |
| 1.610373 | 4.008612 | 4.051178 | 8.25E-05 | 0.001659 | 1.245168 | KISS1R |
| 1.177419 | 8.599908 | 2.684682 | 0.0081 | 0.047763 | -2.89814 | BEX2 |
| 1.284346 | 6.018191 | 5.994762 | 1.53E-08 | 4.96E-06 | 9.282866 | PSRC1 |
| -1.09826 | 3.896985 | -2.98007 | 0.003377 | 0.025338 | -2.12612 | CNDP1 |
| -1.49895 | 9.846449 | -5.86352 | 2.90E-08 | 7.66E-06 | 8.675783 | CYP4F2 |
| -1.3308 | 8.505774 | -3.49516 | 0.000628 | 0.007441 | -0.61263 | RDH16 |
| -1.33882 | 12.92817 | -4.53968 | 1.17E-05 | 0.000417 | 3.054278 | HSD17B6 |
| 1.060487 | 6.749553 | 4.942034 | 2.09E-06 | 0.000128 | 4.658392 | B3GALNT1 |
| -1.25456 | 10.8326 | -5.61464 | 9.63E-08 | 1.67E-05 | 7.547224 | CES2 |
| 2.048183 | 5.09946 | 5.991508 | 1.55E-08 | 4.96E-06 | 9.267717 | HCAR3 |
| 1.150298 | 7.72311 | 3.643441 | 0.000373 | 0.005132 | -0.13919 | VNN2 |
| 1.202973 | 5.066559 | 4.664301 | 6.93E-06 | 0.000299 | 3.540537 | SPHK1 |
| 1.82023 | 5.768316 | 4.142183 | 5.80E-05 | 0.001301 | 1.57011 | HS6ST2 |
| -1.41206 | 4.985012 | -4.09406 | 6.99E-05 | 0.001473 | 1.397563 | CLDN2 |
| -1.11447 | 9.448631 | -5.57184 | 1.18E-07 | 1.94E-05 | 7.356228 | RGN |
| 1.285439 | 8.732567 | 5.13346 | 8.93E-07 | 7.35E-05 | 5.455259 | ZC3HAV1L |
| -1.4522 | 6.230975 | -3.50951 | 0.000597 | 0.007227 | -0.56753 | CD5L |
| -1.07308 | 8.081885 | -3.64997 | 0.000365 | 0.005042 | -0.11798 | IL32 |
| 1.41557 | 5.357487 | 3.901208 | 0.000146 | 0.0025 | 0.722212 | GCNT3 |
| 1.319452 | 6.642474 | 4.797662 | 3.92E-06 | 0.0002 | 4.071514 | TRIP13 |
| 1.454507 | 7.426816 | 3.305808 | 0.001192 | 0.011975 | -1.19305 | FOXQ1 |
| -1.16492 | 7.421156 | -4.25828 | 3.67E-05 | 0.000925 | 1.992822 | ABCG2 |
| -1.02027 | 6.914643 | -4.04092 | 8.58E-05 | 0.001709 | 1.208884 | HOMER2 |
| 1.000542 | 6.939837 | 4.32521 | 2.81E-05 | 0.000771 | 2.240592 | KIF23 |
| 1.239755 | 5.577016 | 4.302197 | 3.08E-05 | 0.000822 | 2.155062 | GAL3ST1 |
| 1.023339 | 5.109323 | 2.666416 | 0.008531 | 0.049411 | -2.94351 | SNCAIP |
| 1.013825 | 7.244536 | 3.924743 | 0.000133 | 0.002338 | 0.803238 | DLGAP5 |
| 1.002213 | 7.120461 | 3.662135 | 0.000349 | 0.004874 | -0.07835 | CDC6 |
| 1.509028 | 7.23338 | 4.890736 | 2.62E-06 | 0.000152 | 4.448448 | CDC20 |
| -1.70832 | 6.520519 | -4.42469 | 1.88E-05 | 0.000576 | 2.614333 | NR1I3 |

**Supplementary Table 2.** More effective drugs in TACE responders.

| drug_name | response_mean | nonresponse_mean | p | adj_p | pathway_name | targets |
| --- | --- | --- | --- | --- | --- | --- |
| AZD2014 | 7.311138 | 10.58625 | 5.24E-07 | 0.000104 | PI3K/MTOR signaling | mTORC1, mTORC2 |
| Nutlin-3a (-) | 35.42027 | 54.72019 | 3.21E-06 | 0.00025 | p53 pathway | MDM2 |
| Phenformin | 1838.311 | 2422.146 | 3.40E-06 | 0.00025 | Other | Biguanide agent |
| MK-2206 | 5.37452 | 6.67587 | 3.03E-05 | 0.001234 | PI3K/MTOR signaling | AKT1, AKT2 |
| Pictilisib | 3.305694 | 4.05696 | 0.000903 | 0.009383 | PI3K/MTOR signaling | PI3K (class 1) |
| Doramapimod | 85.68732 | 93.37421 | 0.000275 | 0.011332 | JNK and p38 signaling | p38, JNK2 |
| Uprosertib | 19.06773 | 25.4427 | 0.000286 | 0.011332 | PI3K/MTOR signaling | AKT1, AKT2, AKT3 |
| JNJ38877605 | 71.33575 | 74.17573 | 0.001324 | 0.011572 | RTK signaling | MET |
| LY2109761 | 166.2347 | 197.3224 | 0.00041 | 0.013526 | Other | TGFB1 |
| Capivasertib | 20.87123 | 28.25648 | 0.001717 | 0.01373 | PI3K/MTOR signaling | AKT |
| Tanespimycin | 0.526413 | 0.664174 | 0.001754 | 0.01373 | Protein stability and degradation | HSP90 |
| Rapamycin | 0.151608 | 0.179827 | 0.00181 | 0.013835 | PI3K/MTOR signaling | MTORC1 |
| XAV939 | 77.82331 | 87.85348 | 0.000612 | 0.015313 | WNT signaling | TNKS1, TNKS2 |
| AZD1208 | 191.3302 | 224.1493 | 0.001095 | 0.021684 | Other, kinases | PIM1, PIM2, PIM3 |
| MN-64 | 108.2324 | 124.549 | 0.001432 | 0.023635 | WNT signaling | TNKS1, TNKS2 |
| Ribociclib | 41.34247 | 47.03536 | 0.001631 | 0.024838 | Cell cycle | CDK4, CDK6 |
| AZD6482 | 22.25675 | 26.65706 | 0.00311 | 0.038484 | PI3K/MTOR signaling | PI3Kbeta |
| Selumetinib | 13.5851 | 15.75835 | 0.007913 | 0.046095 | ERK MAPK signaling | MEK1, MEK2 |

**Supplementary Table 3.** More effective drugs in TACE non-responders.

| drug_name | response_mean | nonresponse_mean | p | adj_p | pathway_name | targets |
| --- | --- | --- | --- | --- | --- | --- |
| NSC-87877 | 170.2313 | 138.1874 | 2.03E-10 | 7.47E-08 | Other | SHP-1 (PTPN6), SHP-2 (PTPN11) |
| Pyrimethamine | 62.95543 | 43.06502 | 4.62E-10 | 8.48E-08 | Other | Dihydrofolate reductase (DHFR) |
| CPI-613 | 193.7716 | 170.4763 | 1.72E-06 | 0.00021 | Other | Metabo, Mitochondria |
| Brivanib, BMS-540215 | 84.1739 | 68.45924 | 4.51E-06 | 0.000276 | RTK signaling | VEGFR, PDGFR |
| ETP-45835 | 206.3261 | 182.8448 | 1.11E-05 | 0.000539 | Other, kinases | MNK1, MNK2 |
| Thapsigargin | 0.015076 | 0.014265 | 1.18E-05 | 0.000539 | Other | SERCA |
| NSC-207895 | 65.17423 | 49.93205 | 4.41E-05 | 0.00162 | p53 pathway | MDM4 |
| Cisplatin | 18.94953 | 15.29262 | 5.19E-05 | 0.001731 | DNA replication | DNA crosslinker |
| Docetaxel | 0.008347 | 0.006392 | 7.24E-05 | 0.002212 | Mitosis | Microtubule stabiliser |
| PF-00299804 | 7.696917 | 6.352105 | 8.11E-05 | 0.002212 | RTK signaling | EGFR, ERBB2, ERBB4 |
| FTI-277 | 8.772655 | 7.925769 | 8.44E-05 | 0.002212 | Other | Farnesyl-transferase (FNTA) |
| Entinostat | 2.429254 | 2.132427 | 9.60E-05 | 0.00228 | Chromatin histone acetylation | HDAC1, HDAC3 |
| AZD4547 | 8.946654 | 7.82264 | 9.94E-05 | 0.00228 | RTK signaling | FGFR1, FGFR2, FGFR3 |
| JQ12 | 4.222431 | 3.042045 | 0.000107 | 0.002303 | Chromatin histone acetylation | HDAC1, HDAC2 |
| PF-562271 | 7.485742 | 6.066923 | 0.000124 | 0.002529 | Cytoskeleton | FAK, FAK2 |
| GSK1904529A | 9.862589 | 9.110153 | 0.000149 | 0.002883 | RTK signaling | IGF1R, IR |
| BI-2536 | 1.659261 | 1.208348 | 3.04E-05 | 0.003012 | Cell cycle | PLK1, PLK2, PLK3 |
| KIN001-042 | 95.12091 | 86.30638 | 0.000228 | 0.004186 | WNT signaling | GSK3B |
| PLK_6522 | 0.1064 | 0.075359 | 0.000251 | 0.004384 | Cell cycle | PLK1, PLK2, PLK3 |
| Vinorelbine | 0.0217 | 0.016985 | 0.000265 | 0.004421 | Mitosis | Microtubule destabiliser |
| Epothilone B | 0.007657 | 0.005883 | 0.000289 | 0.0045 | Mitosis | Microtubule stabiliser |
| UNC0638 | 25.99501 | 21.03623 | 0.000296 | 0.0045 | Chromatin histone methylation | G9a and GLP methyltransferases |
| Temozolomide | 300.2354 | 264.6107 | 0.000307 | 0.0045 | DNA replication | DNA alkylating agent |
| Etoposide | 5.958173 | 4.159491 | 0.000334 | 0.004537 | DNA replication | TOP2 |
| Alectinib | 49.11489 | 43.63868 | 0.000333 | 0.004537 | RTK signaling | ALK |
| IAP_7638 | 64.76952 | 53.1504 | 0.000394 | 0.005165 | Other | IAP |
| GSK1070916 | 8.079563 | 6.100397 | 0.000432 | 0.005461 | Mitosis | AURKA, AURKC |
| Masitinib | 42.50227 | 36.03712 | 0.000469 | 0.005736 | RTK signaling | KIT, PDGFRA, PDGFRB |
| LCL161 | 44.0826 | 39.41192 | 0.000568 | 0.006517 | Apoptosis regulation | XIAP, cIAP1, cIAP2 |
| GSK650394 | 32.17251 | 26.74747 | 0.000592 | 0.006585 | Other, kinases | SGK2, SGK3 |
| Vinblastine | 0.018488 | 0.014359 | 0.000749 | 0.008084 | Mitosis | Microtubule destabiliser |
| FGFR_0939 | 9.624426 | 8.214786 | 0.00092 | 0.009383 | RTK signaling | FGFR4 |
| PHA-793887 | 16.54867 | 11.99487 | 0.001074 | 0.01038 | Cell cycle | CDK2, CDK7, CDK5 |
| GW-2580 | 220.7761 | 203.005 | 0.001135 | 0.010662 | RTK signaling | CSF1R |
| SU11274 | 46.16006 | 40.1924 | 0.001162 | 0.010662 | RTK signaling | MET |
| T0901317 | 91.92046 | 82.34413 | 0.001307 | 0.011572 | Other | LXR, FXR |
| JW-7-24-1 | 4.92439 | 4.164228 | 0.001456 | 0.012423 | Other, kinases | LCK |
| SB216763 | 88.42399 | 78.89536 | 0.00162 | 0.013515 | WNT signaling | GSK3A, GSK3B |
| MetAP2 Inhibitor, A832234 | 11.3508 | 10.01687 | 0.001758 | 0.01373 | Other | MetAP2 |
| FGFR_3831 | 19.69831 | 16.69945 | 0.001853 | 0.013882 | RTK signaling | FGFR1, FGFR2, FGFR3, FGFR4 |
| Pemetrexed | 25.73421 | 20.58283 | 0.001932 | 0.013904 | DNA replication | TYMS |
| GW843682X | 0.093439 | 0.07004 | 0.001925 | 0.013904 | Cell cycle | PLK1 |
| MG-132 | 0.218411 | 0.188904 | 0.000619 | 0.015313 | Protein stability and degradation | Proteasome, CAPN1 |
| MK-1775 | 2.291769 | 1.661156 | 0.000995 | 0.021684 | Cell cycle | WEE1, PLK1 |
| Sepantronium bromide | 0.018466 | 0.013494 | 0.001238 | 0.022285 | Apoptosis regulation | BIRC5 |
| AT-7519 | 2.810608 | 2.146761 | 0.003275 | 0.023116 | Cell cycle | CDK1, CDK2, CDK4, CDK6, CDK9 |
| MIM1 | 53.9063 | 46.43283 | 0.001884 | 0.026218 | Apoptosis regulation | MCL1 |
| GSK429286A | 138.0261 | 117.9404 | 0.003905 | 0.027038 | Cytoskeleton | ROCK1, ROCK2 |
| Dacinostat | 0.058981 | 0.051818 | 0.004048 | 0.027513 | Chromatin histone acetylation | HDAC1 |
| CP724714 | 84.26534 | 76.29558 | 0.004693 | 0.031313 | RTK signaling | ERBB2 |
| PD173074 | 15.3014 | 13.67049 | 0.004798 | 0.031444 | RTK signaling | FGFR1, FGFR2, FGFR3 |
| NPK76-II-72-1 | 22.10047 | 18.10346 | 0.005671 | 0.036516 | Cell cycle | PLK3 |
| Daporinad | 0.015779 | 0.011598 | 0.003349 | 0.03901 | Metabolism | NAMPT |
| Zibotentan | 252.688 | 240.192 | 0.006172 | 0.039054 | Other | Endothelin-1 receptor (EDNRA) |
| XMD13-2 | 34.73657 | 29.48429 | 0.006906 | 0.040878 | Apoptosis regulation | RIPK1 |
| CAY10566 | 22.12641 | 20.06861 | 0.006819 | 0.040878 | Other | Stearoyl-CoA desaturase |
| VX-11e | 22.50337 | 18.37187 | 0.006898 | 0.040878 | ERK MAPK signaling | ERK2 |
| Linsitinib | 10.46502 | 10.02719 | 0.008177 | 0.046889 | IGF1R signaling | IGF1R |

**Supplementary Table 4.** Performance of five models based on 373 DEGs.

| Models | AUC | F1 score | Accuracy | Youden index | Sensitivity | Specificity | PPV | NPV |
| --- | --- | --- | --- | --- | --- | --- | --- | --- |
| Log | 0.900 | 0.790 | 0.817 | 0.631 | 0.808 | 0.824 | 0.786 | 0.856 |
| SVM | 0.900 | 0.790 | 0.800 | 0.613 | 0.854 | 0.759 | 0.742 | 0.872 |
| ANN | 0.895 | 0.786 | 0.807 | 0.615 | 0.815 | 0.800 | 0.766 | 0.852 |
| XGBoost | 0.846 | 0.748 | 0.780 | 0.554 | 0.754 | 0.800 | 0.752 | 0.813 |
| RF | 0.821 | 0.713 | 0.747 | 0.486 | 0.715 | 0.771 | 0.724 | 0.778 |

Log: Lasso-logistic regression; SVM: support vector machine; ANN: artificial neural network; RF: random forest.

**Supplementary Table 5.** Top20 important genes of each model.

| model | Symbol | count | ranking_sum | mean_rank |
| --- | --- | --- | --- | --- |
| ANN | SLAIN1 | 10 | 95 | 9.5 |
|  | VWF | 10 | 107 | 10.7 |
|  | SLC9A3-AS1 | 10 | 116 | 11.6 |
|  | TSPAN8 | 10 | 180 | 18 |
|  | IGF2BP3 | 10 | 211 | 21.1 |
|  | ERAP2 | 10 | 226 | 22.6 |
|  | HERC6 | 10 | 267 | 26.7 |
|  | ADH1C | 10 | 269 | 26.9 |
|  | AQP1 | 10 | 275 | 27.5 |
|  | LOX | 10 | 425 | 42.5 |
|  | CXCL11 | 9 | 138 | 15.3 |
|  | ZC3HAV1L | 9 | 150 | 16.7 |
|  | FABP4 | 9 | 180 | 20 |
|  | DHRS2 | 9 | 189 | 21 |
|  | TYRO3 | 9 | 198 | 22 |
|  | DUSP9 | 9 | 201 | 22.3 |
|  | S100A8 | 9 | 237 | 26.3 |
|  | LARP6 | 9 | 283 | 31.4 |
|  | HMGA2 | 9 | 318 | 35.3 |
|  | EPCAM | 9 | 341 | 37.9 |
| Logistic | SPARCL1 | 10 | 31 | 3.1 |
|  | SLAIN1 | 10 | 49 | 4.9 |
|  | SLC9A3-AS1 | 9 | 32 | 3.6 |
|  | TYRO3 | 9 | 192 | 21.3 |
|  | ADH1C | 9 | 194 | 21.6 |
|  | TSPAN8 | 8 | 25 | 3.1 |
|  | PKIB | 8 | 72 | 9 |
|  | S100A8 | 8 | 80 | 10 |
|  | ZC3HAV1L | 8 | 88 | 11 |
|  | CXCL11 | 8 | 90 | 11.2 |
|  | AQP1 | 8 | 128 | 16 |
|  | TIAM1 | 8 | 138 | 17.2 |
|  | ERAP2 | 8 | 161 | 20.1 |
|  | HERC6 | 8 | 200 | 25 |
|  | LOX | 8 | 227 | 28.4 |
|  | DHRS2 | 7 | 166 | 23.7 |
|  | FABP4 | 7 | 167 | 23.9 |
|  | LIN28B | 7 | 195 | 27.9 |
|  | VWF | 7 | 216 | 30.9 |
|  | SLC35G1 | 7 | 266 | 38 |
| xgb | SPARCL1 | 10 | 101 | 10.1 |
|  | SLAIN1 | 10 | 362 | 36.2 |
|  | VSTM4 | 9 | 174 | 19.3 |
|  | FAS | 9 | 284 | 31.6 |
|  | SLC9A3-AS1 | 9 | 306 | 34 |
|  | KANK4 | 9 | 402 | 44.7 |
|  | OGDHL | 8 | 48 | 6 |
|  | SLC35G1 | 8 | 289 | 36.1 |
|  | ERAP2 | 8 | 352 | 44 |
|  | ADH1C | 7 | 169 | 24.1 |
|  | VWF | 7 | 257 | 36.7 |
|  | HERC6 | 7 | 285 | 40.7 |
|  | ENPP3 | 7 | 366 | 52.3 |
|  | ZNF711 | 6 | 145 | 24.2 |
|  | MMP12 | 6 | 198 | 33 |
|  | FIGN | 6 | 199 | 33.2 |
|  | PPL | 6 | 221 | 36.8 |
|  | HCAR3 | 6 | 231 | 38.5 |
|  | PEG10 | 6 | 238 | 39.7 |
|  | S100A8 | 6 | 255 | 42.5 |
| rf | OGDHL | 9 | 65 | 7.2 |
|  | SPARCL1 | 8 | 62 | 7.8 |
|  | BAMBI | 8 | 155 | 19.4 |
|  | HPD | 8 | 229 | 28.6 |
|  | MMP12 | 8 | 238 | 29.8 |
|  | VSTM4 | 7 | 135 | 19.3 |
|  | HILPDA | 7 | 151 | 21.6 |
|  | LPCAT1 | 7 | 180 | 25.7 |
|  | NUDT6 | 7 | 188 | 26.9 |
|  | SLAIN1 | 7 | 262 | 37.4 |
|  | BTNL9 | 6 | 77 | 12.8 |
|  | PON3 | 6 | 112 | 18.7 |
|  | ZNF711 | 6 | 122 | 20.3 |
|  | GAS2L3 | 6 | 156 | 26 |
|  | IFIT1 | 6 | 159 | 26.5 |
|  | IGF2BP2 | 6 | 171 | 28.5 |
|  | CPED1 | 6 | 174 | 29 |
|  | HK2 | 6 | 201 | 33.5 |
|  | HMGA2 | 6 | 287 | 47.8 |
|  | ABAT | 5 | 67 | 13.4 |
| svc | SLC9A3-AS1 | 10 | 31 | 3.1 |
|  | TSPAN8 | 10 | 31 | 3.1 |
|  | SLAIN1 | 10 | 68 | 6.8 |
|  | SPARCL1 | 10 | 70 | 7 |
|  | PKIB | 10 | 121 | 12.1 |
|  | S100A8 | 10 | 128 | 12.8 |
|  | AQP1 | 10 | 212 | 21.2 |
|  | ERAP2 | 9 | 98 | 10.9 |
|  | TYRO3 | 9 | 135 | 15 |
|  | TIAM1 | 9 | 171 | 19 |
|  | HERC6 | 9 | 224 | 24.9 |
|  | ZC3HAV1L | 9 | 248 | 27.6 |
|  | LOX | 9 | 306 | 34 |
|  | FABP4 | 8 | 178 | 22.2 |
|  | PEG10 | 8 | 194 | 24.2 |
|  | SLC35G1 | 8 | 256 | 32 |
|  | VWF | 7 | 68 | 9.7 |
|  | CXCL11 | 7 | 89 | 12.7 |
|  | DHRS2 | 7 | 93 | 13.3 |
|  | ADH1C | 7 | 103 | 14.7 |
