# Supplementary material for: A 10-Gene Signature Identified by Machine Learning for Predicting the Response to Transarterial Chemoembolization in Patients with Hepatocellular Carcinoma
Source: J Oncol. 2022 Jan 24;2022:3822773. doi: 10.1155/2022/3822773 (PMC8803430; doi:10.1155/2022/3822773)
Supplement: Supplementary Materials — Supplementary Figure 1. Survival prediction efficacy of our model. A, the 1-, 3-, and 5-year time-dependent ROC curve as well as relative AUC assessing the efficacy of our model in predicting OS of patients receiving TACE. B and C, the 1-, 3-, and 5-year time-dependent ROC curve as well as relative AUC assessing the efficacy of our model in predicting OS and RFS of patients receiving adjuvant TACE. D, the 1-, 3-, and 5-year time-dependent ROC curve as well as relative AUC assessing the efficacy of our model in predicting OS of patients receiving postrecurrence TACE. E and F, the 1-, 3-, and 5-year time-dependent ROC curve as well as relative AUC assessing the efficacy of our model in predicting OS and RFS of patients receiving resection only. G, calculated AUC value at any given time points between 10 and 60 months in different patient groups. Supplementary Table 1. DEGs between TACE responders and nonresponders. Supplementary Table 2. More effective drugs in TACE responders. Supplementary Table 3. More effective drugs in TACE nonresponders. Supplementary Table 4. Performance of five models based on 373 DEGs. Supplementary Table 5. Top 20 important genes of each model. [file 3822773.f1.zip › 3822773.f1/Supplementary Figure 1.docx]

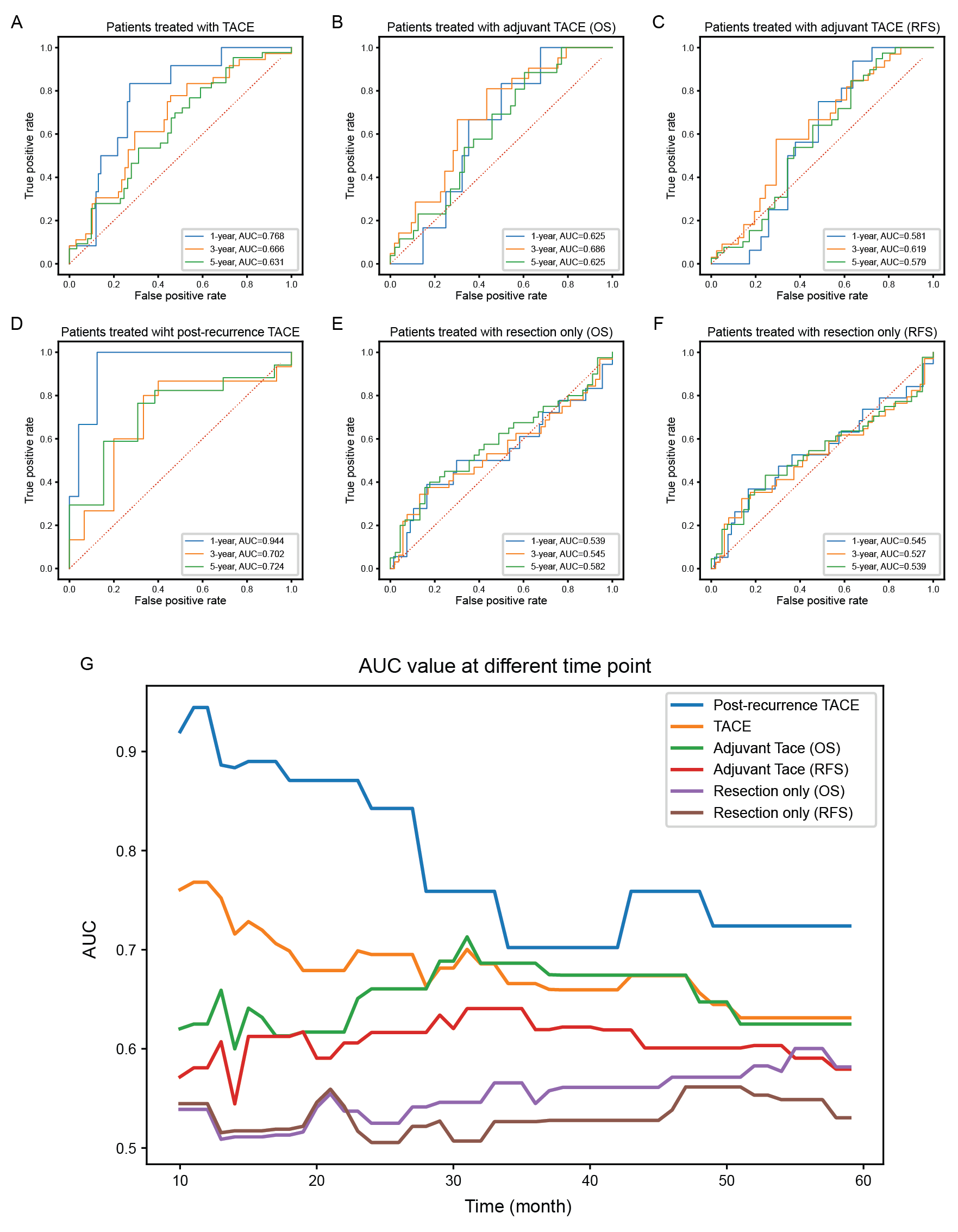


**Supplementary Figure 1.** Survival prediction efficacy of our model. A, The 1-, 3- and 5-year time-dependent ROC curve as well as relative AUC assessing the efficacy of our model in predicting OS of patients receiving TACE. B, The 1-, 3- and 5-year time-dependent ROC curve as well as relative AUC assessing the efficacy of our model in predicting OS of patients receiving adjuvant TACE. C, The 1-, 3- and 5-year time-dependent ROC curve as well as relative AUC assessing the efficacy of our model in predicting RFS of patients receiving adjuvant TACE. D, The 1-, 3- and 5-year time-dependent ROC curve as well as relative AUC assessing the efficacy of our model in predicting OS of patients receiving post-recurrence TACE. E, The 1-, 3- and 5-year time-dependent ROC curve as well as relative AUC assessing the efficacy of our model in predicting OS of patients receiving resection only. F, The 1-, 3- and 5-year time-dependent ROC curve as well as relative AUC assessing the efficacy of our model in predicting RFS of patients receiving resection only. G, Calculated AUC value at any given time points between 10-60 months in different patient groups.
